# Early programming of the oocyte epigenome temporally controls late prophase I transcription and chromatin remodelling

**DOI:** 10.1038/ncomms12331

**Published:** 2016-08-10

**Authors:** Paulo Navarro-Costa, Alicia McCarthy, Pedro Prudêncio, Christina Greer, Leonardo G. Guilgur, Jörg D. Becker, Julie Secombe, Prashanth Rangan, Rui G. Martinho

**Affiliations:** 1 Departamento de Ciências Biomédicas e Medicina, and Center for Biomedical Research, Universidade do Algarve, Campus de Gambelas, 8005-139 Faro, Portugal; 2 Instituto Gulbenkian de Ciência, 2780-156 Oeiras, Portugal; 3 Department of Biological Sciences/RNA Institute, University at Albany SUNY, Albany, New York 12222, USA; 4 Department of Genetics, Albert Einstein College of Medicine, Bronx, New York 10461, USA

## Abstract

Oocytes are arrested for long periods of time in the prophase of the first meiotic division (prophase I). As chromosome condensation poses significant constraints to gene expression, the mechanisms regulating transcriptional activity in the prophase I-arrested oocyte are still not entirely understood. We hypothesized that gene expression during the prophase I arrest is primarily epigenetically regulated. Here we comprehensively define the *Drosophila* female germ line epigenome throughout oogenesis and show that the oocyte has a unique, dynamic and remarkably diversified epigenome characterized by the presence of both euchromatic and heterochromatic marks. We observed that the perturbation of the oocyte's epigenome in early oogenesis, through depletion of the dKDM5 histone demethylase, results in the temporal deregulation of meiotic transcription and affects female fertility. Taken together, our results indicate that the early programming of the oocyte epigenome primes meiotic chromatin for subsequent functions in late prophase I.

## Abstract (web summary)

Meiotically arrested oocytes retain transcriptional ability despite chromosome condensation. Here, the authors show that *Drosophila* oocytes regulate meiotic transcription and chromatin remodelling through the programming, in early oogenesis, of an extremely diversified epigenome.

## Graphical abstract

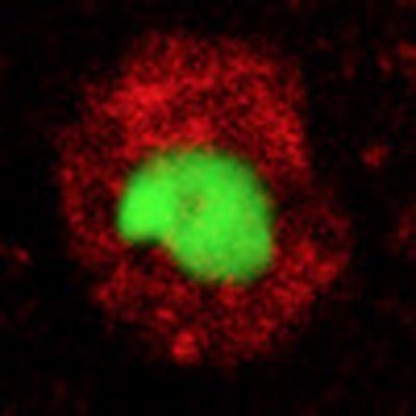

Oocytes remain arrested for a significant amount of time during prophase of the first meiotic division (prophase I). This prophase I arrest is remarkably conserved across Metazoans and is essential for oocyte differentiation^1^. Prophase I chromosomes are organized as bivalents—pairs of homologous chromosomes connected by chiasmata and sister chromatid cohesion^2^. The formation of bivalents requires chromosome condensation and compaction, both of which pose significant constraints to gene expression^3^,^4^. Such constraints are particularly problematic in the context of female meiosis, because during the prolonged prophase I arrest the oocyte needs to accumulate maternal components that are essential for oogenesis and early embryogenesis.

Oocytes have developed distinct strategies to ensure gene expression while maintaining chromosome condensation^5^. In the case of insects that undergo meroistic oogenesis, such as *Drosophila*, germ line transcriptional activity is ensured by the nurse cells—non-meiotic germ cells in cytoplasmic continuity with the oocyte^6^,^7^. Nurse cells sustain oocyte development via the synthesis and transport of maternal factors into the developing female gamete. Accordingly, the differentiation of nurse cells allows the *Drosophila* oocyte to shutdown transcription throughout most of oogenesis^8^. This oocyte transcriptional quiescence is associated with the reorganization of the oocyte's chromatin into a highly compact cluster of meiotic chromosomes referred to as the karyosome^9^,^10^. Surprisingly, despite being transcriptionally inactive throughout most of the prophase I arrest, cytological findings from the early 1970s indicate that the *Drosophila* oocyte reactivates transcription prior to the resumption of meiosis^11^. This often-overlooked observation poses two fundamental questions: how is transcription possible in such highly compacted chromatin and what is the functional relevance of oocyte transcription for meiotic progression?

Our results indicate that the programming of the *Drosophila* oocyte epigenome during early oogenesis controls, several hours later, the transcriptional reactivation of meiotic chromatin. In this regard, we find that the oocyte epigenome is unique, being remarkably diversified and dynamic in terms of euchromatic and heterochromatic marks. We show that the disruption of the oocyte epigenome, mainly through increased levels of histone H3 lysine 4 trimethylation (H3K4me3), a euchromatic mark associated with the transcription start site of active genes^12^,^13^, leads to significant defects in three main biological programs: (i) temporal control of gene expression; (ii) regulation of RNA polymerase II (RNAPII) levels in oocyte chromatin; and (iii) remodelling of the meiotic chromosomes in late prophase I. The nature of these defects has a critical impact on meiotic completion and female fertility.

## Results

### *Drosophila* oocytes reactivate transcription during meiosis

**Figure 1:**
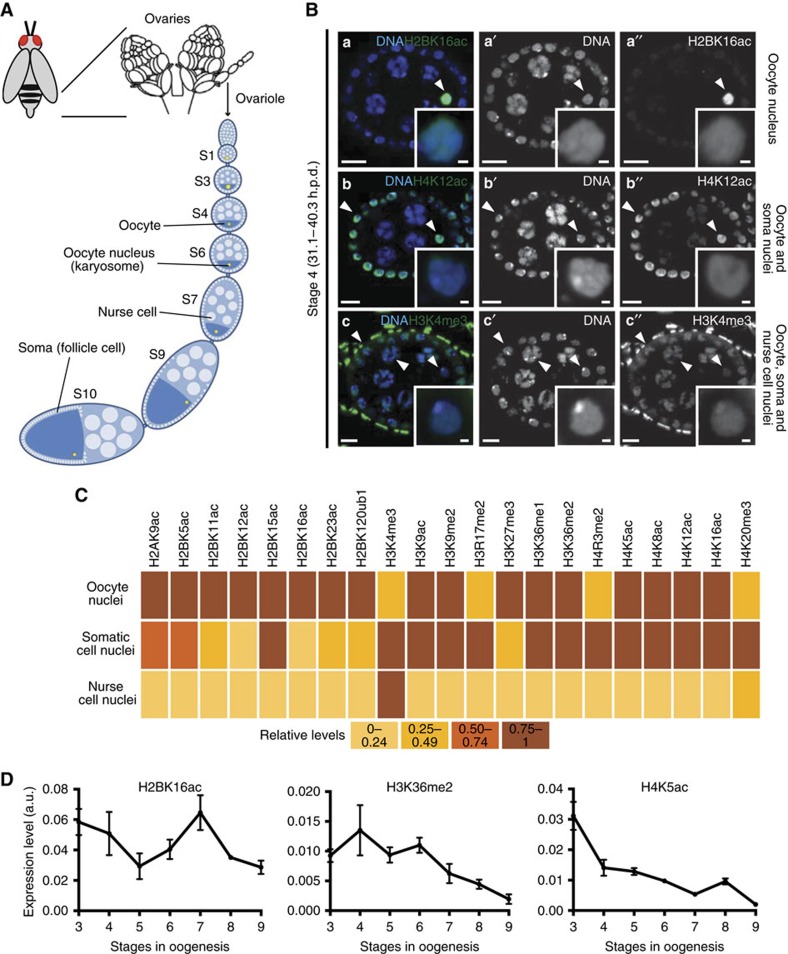
*Drosophila* oocytes have a unique, dynamic and diversified epigenome. (**A**) Schematic of *Drosophila* oogenesis. The functional unit of the *Drosophila* ovary is the ovarian follicle or egg chamber, which consists of the cyst defined by the oocyte and its supporting nurse cells surrounded by a monolayer of somatic cells (follicle cells). The morphological features of the ovarian follicles define 14 distinct developmental stages (S). The oocyte progresses through the initial phases of prophase I until it arrests at diplotene at oogenesis stage 5. The onset of this arrest is preceded by the clustering of oocyte chromosomes into a highly compact and transcriptionally quiescent structure—the karyosome. As oogenesis progresses the oocyte grows in size mainly through cytoplasmic transfer from the nurse cells. At stage 9 the oocyte reactivates transcription, which lasts until the start of stage 11. The prophase I arrest is lifted at stage 13 and results in the formation of a metaphase I-arrested gamete. (**B**) Distinct patterns of histone post-translation modifications (PTMs) in the *Drosophila* ovarian follicle. Three main patterns were identified: (i) oocyte-specific expression (histone H2B lysine 16 acetylation—H2BK16ac; **a**–**a′′**), (ii) oocyte+somatic (follicle) cell expression (histone H4 lysine 12 acetylation—H4K12ac; **b**–**b′′**), and generalized distribution (oocyte (albeit sometimes at lower levels)+nurse cells+somatic cells; histone H3 lysine 4 trimethylation—H3K4me3; **c**–**c′′**). Arrowheads point to the chromatin of different cell types, insets depict oocyte chromatin. Development time in relation to the start of oogenesis is expressed in hours post-germ line stem cell division (h.p.d.). Scale bars, 10 μm for ovarian follicles and 1 μm for oocyte insets. (**C**) Heatmap representing the expression of 21 different histone PTMs across the three different cell types of the *Drosophila* ovarian follicle. (**D**). Temporal analysis of the highly dynamic levels of oocyte H2BK16ac, histone H3 lysine 36 dimethylation (H3K36me2) and histone H4 lysine 5 acetylation (H4K5ac) throughout oogenesis. Relative levels are expressed in fluorescence arbitrary units (a.u.). Error bars represent s.d. See Supplementary Fig. 2 for illustrative micrographs and complete temporal analysis of the tested PTMs.

Consistent with previous reports^11^, we observed using a ethynyl uridine (EU) incorporation assay that the *Drosophila* oocyte is transcriptionally inactive throughout most of the prophase I arrest (from oogenesis stage 5 until the end of stage 8; Fig. 1A, Supplementary Fig. 1). This transcriptional quiescence starts from the onset of the prophase I arrest, lasts for ∼25 h, and is associated with the reorganization of oocyte chromatin into a highly compact cluster of meiotic chromosomes referred to as the karyosome. Despite the prolonged transcriptional inactivity, we observed that *Drosophila* oocytes reactivate gene expression ∼13 h before meiotic resumption (at oogenesis stage 9). This precisely-timed oocyte transcriptional reactivation is intriguing, as the polyploid nurse cells ensure essentially all transcriptional activity in the *Drosophila* female germ line. Such observation raises the possibility that successful meiotic progression requires oocyte-specific transcription during the prophase I arrest.

### *Drosophila* oocytes have a diversified and dynamic epigenome

**Figure 2:**
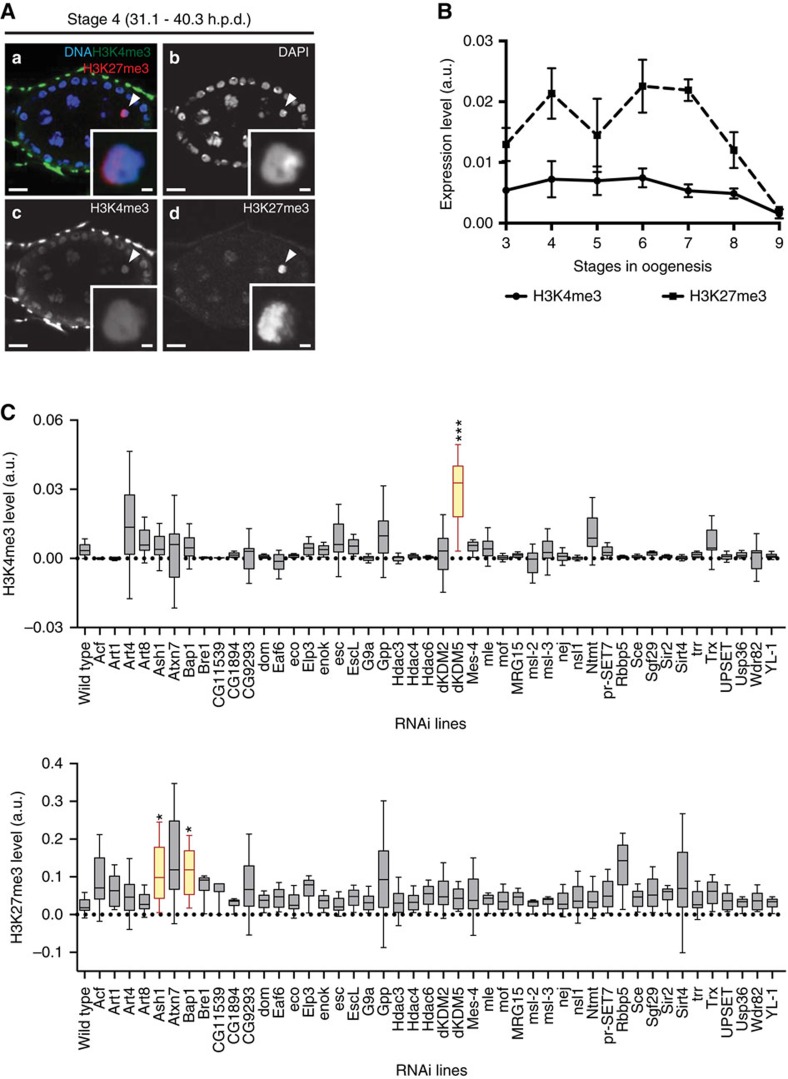
A germ line-specific *in vivo* RNAi screen identified three major regulators of the oocyte epigenome. (**A**) The *Drosophila* oocyte has both activating (histone H3 lysine 4 trimethylation—H3K4me3) and repressive (histone H3 lysine 27 trimethylation—H3K27me3) marks (**a**–**d**). Development time in relation to the start of oogenesis is expressed in hours post-germ line stem cell division (h.p.d.). Arrowheads and insets indicate oocyte chromatin. Scale bars, 10 μm for ovarian follicles and 1 μm for oocyte insets. (**B**) Temporal analysis of oocyte H3K27me3 and H3K4me3 levels throughout oogenesis. The high levels of H3K27me3 during the prophase I arrest are marked by a reduction prior to the onset of oocyte transcriptional reactivation (stage 9). H3K4me3 levels are kept at a low level throughout prophase I. Histone post-translation modifications (PTMs) relative levels are expressed in fluorescence arbitrary units (a.u.). Error bars represent the s.e.m. A total of six replicates were analysed for each data point. (**C**) Effects of the RNAi-mediated knockdown of different chromatin remodellers on oocyte H3K4me3 (top) and H3K27me3 (bottom) levels. A total of 46 different chromatin remodellers were compatible with the development to mid-oogenesis when depleted specifically in the germ line. Of these, three (dKDM5, Ash1 and Bap1) introduced significant disturbances to the oocyte epigenome. Relative histone PTM signals are expressed in fluorescence arbitrary units (a.u.), horizontal lines specify median values, error bars represent s.d. and asterisks indicate significant difference (paired *t*-test; *P*<0.04).

Because of the highly compacted nature of the karyosome-clustered meiotic chromosomes, we hypothesized that *Drosophila* oocyte transcriptional reactivation is regulated by a specialized chromatin state. Indeed, specialized chromatin states have been previously associated with context-specific gene expression requirements in mammals, as in the case of developing male germ cells and of the early embryo^14^,^15^,^16^. To test our hypothesis we set out to define the *Drosophila* oocyte epigenome. In light of the currently insurmountable technical constraints in isolating *Drosophila* oocyte nuclei for chromatin immunoprecipitation-based assays, we took advantage of the spatiotemporal resolution of immunofluorescence to understand not only the epigenetic composition of the oocyte, but also how it can vary throughout oogenesis. Thus, we have established what is currently the most comprehensive map of the *Drosophila* oocyte epigenome through the analysis of 21 histone post-translation modifications (PTMs) in both female germ cells and in the soma. Unexpectedly, we observed that oocyte chromatin has an extremely diversified repertoire of histone PTMs prior to the onset of the prophase I arrest: at oogenesis stage 4, 17 out of the 21 tested histone PTMs were present at high levels in the oocyte (Fig. 1B,C). This result was intriguing not only in light of the cell cycle arrest of the oocyte but also of its impending transcriptional quiescence. Equally noteworthy was the fact that the oocyte epigenome was remarkably dynamic during prophase I, as illustrated by fluctuations in the levels of several histone PTMs during the transcriptionally inactive state (Fig. 1D; Supplementary Fig. 2). Similar to mammals^17^, *Drosophila* oocytes had both euchromatic and heterochromatic marks (Fig. 1C). In particular, we identified in the *Drosophila* oocyte both H3K4me3 (a euchromatic mark) and histone H3 lysine 27 trimethylation (H3K27me3; Fig. 2A), a heterochromatic mark associated with Polycomb-mediated gene repression^18^. We observed that oocyte H3K27me3 levels were dynamically regulated throughout prophase I: the high levels maintained during transcriptional quiescence were decreased as the oocyte reacquired transcriptional activity (at stage 9; Fig. 2B). On the contrary, H3K4me3 levels were kept low and fairly constant throughout prophase I.

### The histone demethylase dKDM5 remodels oocyte H3K4me3

To test if the oocyte's specialized epigenetic state was a critical regulator of transcriptional activity in highly condensed meiotic chromosomes, we decided to selectively disturb the oocyte's epigenome. To do this, we conducted a germ line-specific *in vivo* RNA interference (RNAi) screen with the purpose of identifying enzymes capable of regulating oocyte chromatin. With this technique we specifically depleted throughout oogenesis 50 proteins with known or predicted chromatin-remodelling activity (Supplementary Table 1), and recorded the outcome of such depletion in terms of oocyte H3K4me3 and H3K27me3 levels. Out of the 46 knockdowns that were compatible with development up to mid-oogenesis, we identified three chromatin remodellers able to significantly perturb the levels of either H3K4me3 or H3K27me3 in the oocyte: dKDM5, Ash1 and Bap1 (Fig. 2C).

**Figure 3:**
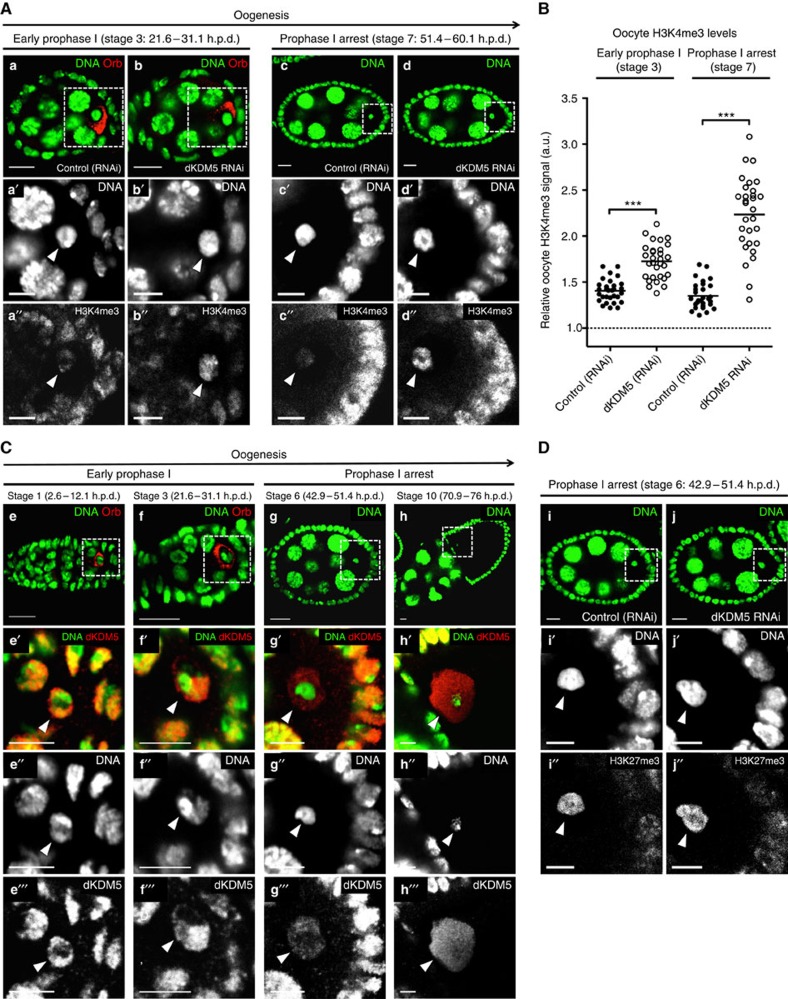
Oocyte H3K4me3 levels are specified in early oogenesis by the histone demethylase dKDM5. (**A**,**B**) Germ line-specific knockdown of the histone demethylase dKDM5 significantly increases histone H3 lysine 4 trimethylation (H3K4me3) levels in prophase I oocytes (**a**–**d′′**). Oocyte H3K4me3 levels were compared at stages 3 and 7 of oogenesis (before and after the establishment of the prophase I arrest, respectively). Signal quantification (**B**) is represented per oocyte and is expressed in fluorescence arbitrary units (a.u.). Horizontal lines specify mean values and asterisks indicate significant difference (Mann–Whitney *U*-test; *P*<0.0001). Similar results were obtained with a *dkdm5*^*−/−*^ mutant (Supplementary Fig. 4A,B). (**C**) dKDM5 is evicted from oocyte chromatin during early oogenesis, prior to the establishment of the prophase I arrest (**e**–**h′′′**) At the very start of prophase I (oogenesis stage 1), dKDM5 co-localizes with oocyte chromatin (**e**–**e′′′**) As the oocyte's chromosomes begin to cluster together to form the karyosome (oogenesis stage 3), dKDM5 is partially evicted from chromatin (**f**–**f′′′**) During the prophase I arrest (oogenesis stage 6 as representative example) dKDM5 does not co-localize with oocyte chromatin, being restricted to the nucleoplasm (**g**–**g′′′**) dKDM5 remains evicted during the transcriptional reactivation of the oocyte in late prophase I (oogenesis stage 10; **h–h′′′**). See Supplementary Fig. 9 for quantification. The dKDM5 signal corresponds to a genomic *dkdm5* transgene containing a C-terminal human influenza hemagglutinin (HA) tag crossed into the *dkdm5*^*−/−*^ mutant background (antibody: anti-HA). (**D**) Oocyte heterochromatin is not globally affected by the germ line-specific knockdown of dKDM5. Heterochromatin of both the facultative (histone H3 lysine 27 trimethylation—H3K27me3) and constitutive (histone H3 lysine 9 dimethylation—H3K9me2) type remained unchanged after dKDM5 knockdown (**i**–**j′′** and Supplementary Fig. 6). Quantifications of oocyte H3K27me3 and H3K9me2 levels are shown in Supplementary Fig. 6. (**A**–**D**) Development time in relation to the start of oogenesis is expressed in hours post-germ line stem cell division (h.p.d.). Rectangles delimit the area of the oocyte insets and arrowheads point to the oocyte chromatin/nucleus. To distinguish early prophase I oocytes from other germ line cells, an oocyte cytoplasm-specific staining was performed (Orb). Scale bars, 10 μm for ovarian follicles and 5 μm for oocyte insets.

Of particular interest to us was the *Drosophila* histone demethylase KDM5 (dKDM5, also known as LID^19^), as its germ line-specific depletion (Supplementary Fig. 3) drastically increased H3K4me3 levels in prophase I oocytes without affecting the levels of the heterochromatic markers H3K27me3 and histone H3 lysine 9 dimethylation (H3K9me2; Fig. 3A,B,D; Supplementary Fig. 4A,B; Supplementary Fig. 5; and Supplementary Fig. 6). The increase in H3K4me3 seen in dKDM5-depleted oocytes was equivalent to that observed in somatic dKDM5 mutant clones (Supplementary Fig. 7), further illustrating the functional relevance of the histone demethylase activity of this enzyme^20^,^21^. In light of their severely disrupted epigenome, we selected dKDM5-depleted oocytes to better understand the specific epigenetic requirements of prophase I-arrested meiotic chromatin.

### dKDM5 temporally regulates meiotic transcription

dKDM5 is a strict H3K4me3 demethylase belonging to the highly conserved family of Jumonji (JmjC) domain-containing proteins^20^,^21^,^22^,^23^. dKDM5 localizes to the transcription start site of developmentally controlled genes, where it has been shown to regulate H3K4me3 levels^24^. While mammalian oocytes have been shown to be particularly sensitive to decreased H3K4me3 levels^25^, the consequences of excessive levels of this modification on female reproductive function remain largely unexplored.

**Figure 4:**
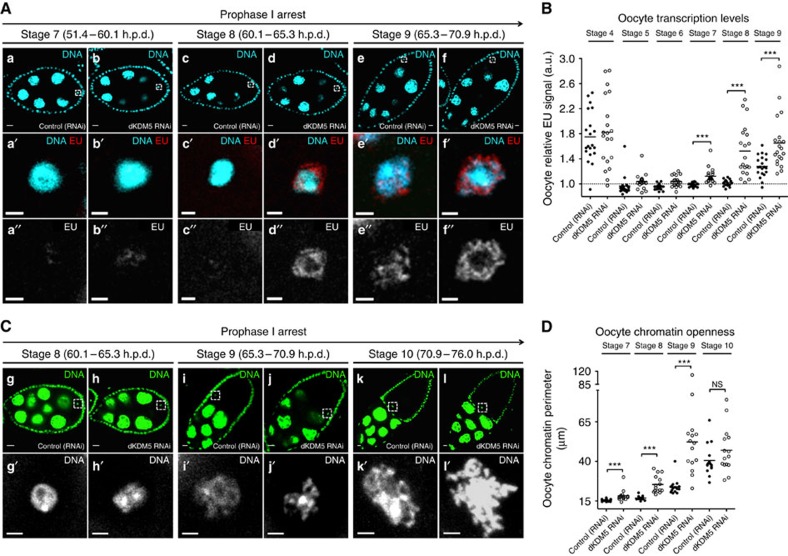
dKDM5 temporally regulates the reactivation of transcription and chromatin remodelling during the prophase I arrest. (**A**,**B**) The germ line-specific knockdown of the histone demethylase dKDM5 results in the premature transcriptional reactivation of prophase I-arrested oocytes (**a**–**f′′**) Oocyte transcription was robustly detected approximately 14 h earlier in dKDM5-depleted conditions when compared with controls: oogenesis stage 7 versus stage 9, respectively. Oocyte transcription was measured by incorporation of the modified nucleotide ethynyl uridine (EU). The specificity of this assay for nascent RNA was confirmed by treatment with the transcription inhibitor Actinomycin D (Supplementary Fig. 13). Signal quantification (**B**) is represented per oocyte and is expressed in fluorescence arbitrary units (a.u.). Horizontal lines specify mean values and asterisks indicate significant difference (Mann–Whitney *U*-test; *P*≤0.0005). Similar results were obtained with a *dkdm5*^*−/−*^ mutant (Supplementary Fig. 4C,D). (**C**,**D**) Meiotic chromosome compaction is remodelled during transcriptional reactivation (**g**–**l′**). Under control conditions, the karyosome-compacted meiotic chromosomes resolve into chromatin protrusions for a period of ∼11 h in late prophase I (from oogenesis stages 9 to 11). dKDM5 knockdown doubled this period to ∼20 h by inducing karyosome opening starting from stage 7 and introduced significant morphological abnormalities to the chromatin (**j′**,**l′**). The perimeter of oocyte chromatin (in μm) was measured for phenotypic quantification of chromatin openness (see **D**). Horizontal lines specify mean values, asterisks indicate significant difference and ‘NS' no significant difference (Mann–Whitney *U*-test; *P*≤0.0002). Similar results were obtained with a *dkdm5*^*−/−*^ mutant (Supplementary Fig. 4I,J). (**A**–**D**) Development time in relation to the start of oogenesis is expressed in hours post-germ line stem cell division (h.p.d.). Rectangles delimit the area of the oocyte insets. Scale bars, 10 μm for ovarian follicles and 2 μm for oocyte insets.

We observed that the germ line-specific depletion of dKDM5 resulted in premature reactivation of gene expression in otherwise transcriptionally quiescent oocytes (Fig. 4A,B; Supplementary Fig. 4C,D). More specifically, upon dKDM5 depletion, prophase I-arrested oocytes anticipated transcriptional reactivation by approximately 14 h (oocyte transcription in dKDM5-depleted conditions was robustly detected at oogenesis stage 7 instead of at stage 9). This premature reactivation of gene expression during the prophase I arrest was not associated with an earlier incapability of these oocytes transiting to the transcriptionally quiescent state. In accordance, dKDM5-depleted oocytes were still able to markedly lower their transcriptional activity at the onset of the prophase I arrest (Fig. 4B), as well as to compact their chromosomes into the karyosome. Such observations further support the hypothesis that H3K27me3 is one of the main drivers for oocyte transcriptional quiescence (Fig. 2B). Analysis of additional histone PTMs revealed that the germ line-specific depletion of dKDM5 could also affect other histone markers of active transcription (Supplementary Fig. 8). More specifically, we observed a decrease in the levels of oocyte histone H3 lysine 9 acetylation (H3K9ac) following dKDM5 depletion (Supplementary Fig. 8A,B). This seemingly counter-intuitive observation is consistent with the previously published data^26^ and likely reflects the complex functional crosstalk between KDM5 and other chromatin remodelling enzymes^27^,^28^. In this regard, we failed to detect any changes in oocyte histone H3 lysine 36 dimethylation (H3K36me2) levels—another histone marker of active transcription (Supplementary Fig. 8C,D). Overall, these data suggest that dKDM5, through its effect on the oocyte epigenome, is a temporal controller of the onset of oocyte transcription during the prophase I arrest.

### dKDM5 specifies oocyte H3K4me3 levels during early oogenesis

To understand how dKDM5 could temporally regulate transcription in late prophase I, we characterized the distribution of this demethylase specifically in oocyte chromatin. Rather surprisingly, we found that dKDM5 was evicted from the oocyte's chromatin at the initial stages of oogenesis, on compaction of the meiotic chromosomes into the karyosome (Fig. 3C; Supplementary Fig. 9). Considering that H3K4me3 levels are kept fairly constant throughout the prophase I arrest (Fig. 2B), the early eviction of dKDM5 from oocyte chromatin strongly suggests that oocyte H3K4me3 levels are specified at the initial stages of oogenesis. Consistent with this, we observed that on dKDM5 depletion, oocyte H3K4me3 levels were already upregulated before the establishment of the prophase I arrest (Fig. 3A,B).

On the basis of these observations, we conclude that dKDM5 acts at the initial stages of oogenesis to prevent excessive levels of H3K4me3 in the oocyte. Furthermore, we propose that dKDM5 is able to temporally regulate the transcriptional reactivation of prophase I-arrested oocytes through the early programming of the oocyte's epigenome.

### dKDM5 determines RNAPII levels during the prophase I arrest

**Figure 5:**
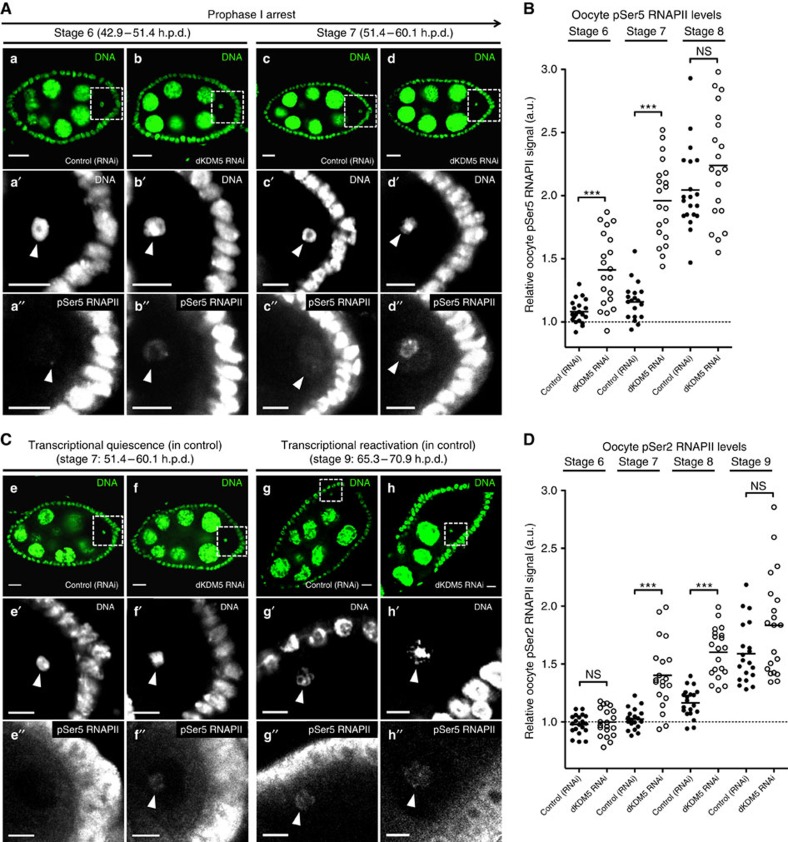
dKDM5 determines the levels of RNA polymerase II in prophase I-arrested chromatin. (**A**,**B**) The germ line-specific knockdown of the histone demethylase dKDM5 significantly increases the levels of RNA polymerase II phosphorylated at position serine 5 of the C-terminal repeat domain (pSer5 RNAPII) in the chromatin of prophase I-arrested oocytes (**a**–**d′′**). (**C**,**D**) Significantly higher levels of transcription elongation (active RNAPII) are observed during the precocious transcriptional reactivation of dKDM5-depleted oocytes (oogenesis stages 7 and 8: **e**–**f′′**; compare with transcriptionally reactivated oocytes in **g**–**h′′**). Active polymerase corresponds to RNAPII phosphorylated at position serine 2 of the C-terminal repeat domain: pSer2 RNAPII. Signal quantification (**B**,**D**) is represented per oocyte and is expressed in fluorescence arbitrary units (a.u.). Horizontal lines indicate mean values, asterisks indicate significant difference and ‘NS' no significant difference (Mann–Whitney *U*-test; *P*<0.0001). Similar results were obtained with a *dkdm5*^*−/−*^ mutant (Supplementary Fig. 4E–H). (**A**–**D**) Development time in relation to the start of oogenesis is expressed in hours post-germ line stem cell division (h.p.d.). Rectangles delimit the area of the oocyte insets, arrowheads point to the oocyte's chromatin. Scale bars, 10 μm for ovarian follicles and 5 μm for oocyte insets.

How can oocyte H3K4me3 levels regulate transcriptional reactivation during the long prophase I arrest? In mammalian cells, H3K4me3 has been shown to directly regulate the assembly of the transcription pre-initiation complex^29^,^30^. This large complex is essential for transcription, as it positions RNAPII on promoters^31^. The phosphorylation state of the C-terminal domain of RNAPII modulates the activity of this polymerase: serine 5 phosphorylation (pSer5) is indicative of a promoter-poised, non-transcribing RNAPII, whereas serine 2 phosphorylation (pSer2) is characteristic of the transcription elongation state^32^. The larger H3K4me3 domains of mammalian cells have been associated with enrichment in both pSer5 and pSer2 RNAPII (ref. 33). Analogously, following germ line-specific dKDM5 depletion, we observed a significant increase in the association of both pSer5 and pSer2 RNAPII to the chromatin of prophase I-arrested oocytes (Fig. 5; Supplementary Fig. 4E–H). Despite the current technical constraints in performing oocyte-specific chromatin immunoprecipitation, our results suggest a mechanistic link between oocyte H3K4me3 levels and the onset of RNAPII-dependent transcriptional reactivation in prophase I-arrested oocytes.

### Meiotic transcription coincides with chromatin remodelling

In accordance with the previous observations^11^, we found that the final stages of the prophase I arrest coincide with a significant remodelling of the karyosome-clustered meiotic chromosomes. More specifically, we observed in late prophase I the opening of the compact karyosome into long chromatin protrusions for a period of ∼11 h (from oogenesis stages 9 to 11; Fig. 4C,D). This remodelling of the meiotic chromosomes is most likely associated with the oocyte transcriptional reactivation that starts at stage 9. Although a causal relationship between the two events remains to be demonstrated, we found that the premature gene expression reactivation seen under dKDM5-depleted conditions doubled the karyosome remodelling period to about 20 h, starting from stage 7 (Fig. 4C,D; Supplementary Fig. 4I,J). This precocious remodelling was temporally coincident with the premature increase in RNAPII levels and mRNA synthesis observed on dKDM5 depletion, suggesting a functional interdependence between the two processes. In addition, meiotic chromatin remodelling was also morphologically abnormal in dKDM5-depleted conditions, as illustrated by overly developed chromatin protrusions (Fig. 4C, see micrographs i′ versus j′ and k′ versus l′). This observation further supports the functional requirement of dKDM5 for correct late prophase I chromosome architecture.

### dKDM5 is required for meiotic completion and fertility

**Figure 6:**
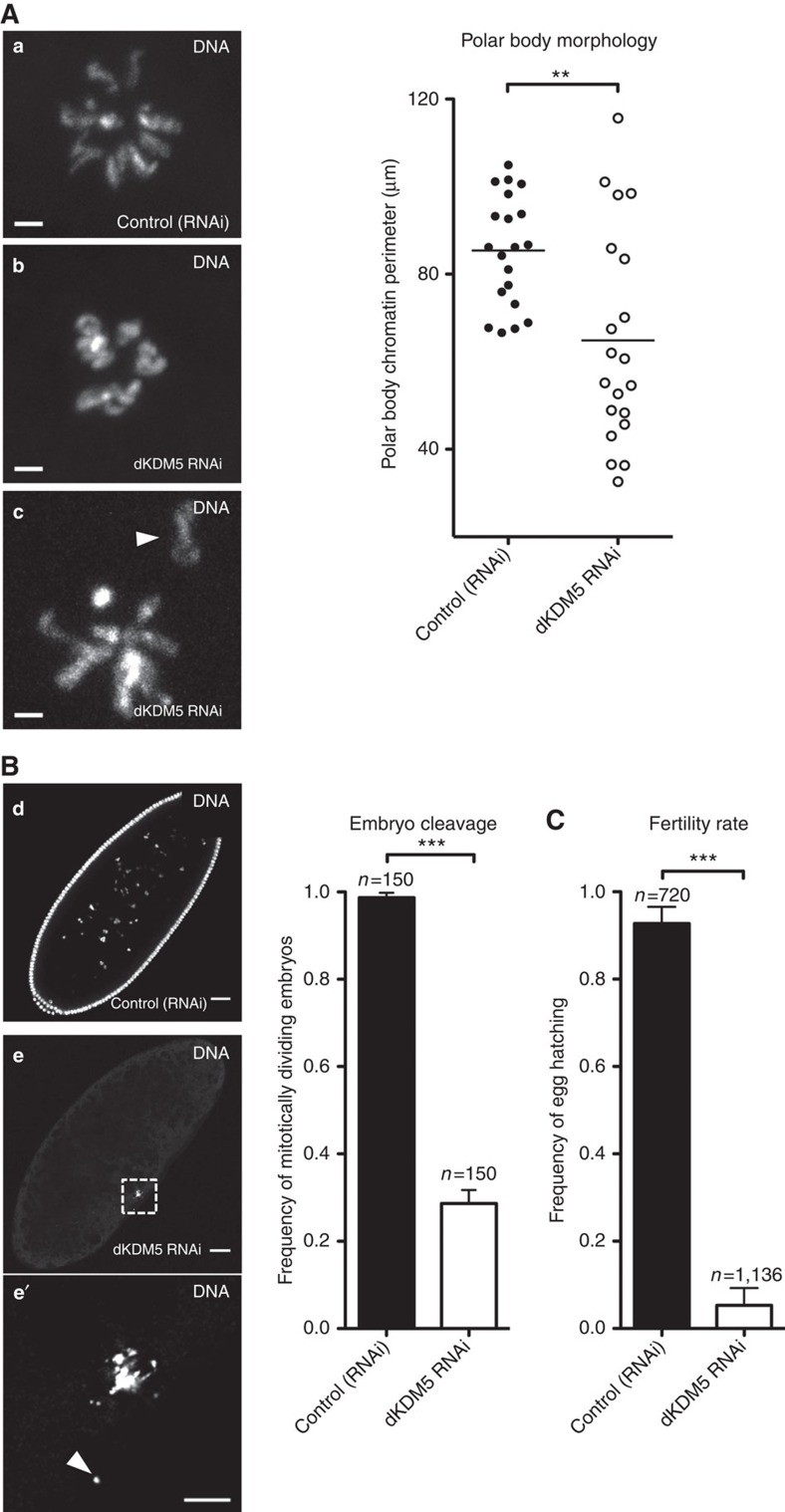
dKDM5 is required for meiotic completion and female fertility. (**A**) dKDM5 is required for correct meiosis. Polar body morphology was used as read-out for successful meiotic completion. Under normal conditions, polar body chromosomes are arranged in a characteristic rosette conformation (**a**). dKDM5 knockdown resulted in significant deviations to this conformation, with scattered chromatin (arrowhead) and defects in chromosome condensation (**b**,**c**). The perimeter of polar body chromatin (in μm) was measured for phenotypic quantification. Horizontal lines specify mean values and asterisks indicate significant difference (Mann–Whitney *U*-test; *P*=0.0031). (**B**) dKDM5-depleted eggs largely fail to initiate mitotic divisions after fertilization (**d**–**e′**). The chromatin of such embryos was scattered (arrowhead in chromatin inset **e′**) and disorganized despite normal gamete size and dorsal–ventral patterning (Supplementary Fig. 11). Error bars represent s.d. and asterisks indicate significant difference (Student's *t*-test; *P*<0.0001). (**C**) Germ line-specific depletion of dKDM5 significantly impairs *Drosophila* fertility. Fertility rate is defined by the frequency of egg hatching 48 h post oviposition. Error bars represent s.d. and asterisks indicate significant difference (Student's *t*-test; *P*<0.0001). (**A**–**C**) Scale bars in **a**–**c**, 2 μm; in **d** and **e**, 30 μm and in **e′**, 10 μm.

In light of the late prophase I chromatin remodelling defects observed after dKDM5 depletion, we investigated to what extent they could impact the successful conclusion of meiosis. Upon completion of the two female meiotic divisions, only one out of the four resulting haploid nuclei gives rise to the female pronucleus^34^. The remaining nuclei, each referred to as a polar body nucleus, eventually degenerate. In *Drosophila*, the three polar bodies nuclei fuse in the common cytoplasm of the egg forming a single polar body with a characteristic rosette pattern^35^,^36^ (Fig. 6A, see micrograph a). The formation of this characteristic polar body is indicative of successful meiotic completion. We found that dKDM5 depletion severely affected meiotic completion, as illustrated by substantial defects in polar body integrity (scattered chromatin and abnormal chromosome condensation; Fig. 6A, see micrographs b and c). In agreement with their significant meiotic defects, dKDM5-depleted eggs also largely failed to enter embryogenesis after fertilization (Fig. 6B). This defect was manifested by a significant fraction of fertilized eggs that were incapable of initiating mitotic divisions. Consequently, we observed that the depletion of dKDM5 during oogenesis was associated with a marked reduction of female fertility (Fig. 6C).

Despite the severely compromised meiotic completion recorded under dKDM5-depleted conditions, we failed to detect noticeable defects in the metaphase I (MI) arrest of mature, non-activated eggs (Supplementary Fig. 10). Our observation is consistent with the previously published data^37^, and suggests that the chromatin morphological disturbances seen under dKDM5-depleted conditions, although critical for meiotic completion, are not sufficient to visibly impair chromosome compaction into the MI arrest. Also noteworthy was the fact that dKDM5-depleted eggs had normal size and dorsal–ventral patterning (Supplementary Fig. 11). The absence of dorsal–ventral patterning defects (as inferred from the correctly differentiated dorsal appendages) in dKDM5-depleted eggs indicates that the DNA damage checkpoint was not activated during early oogenesis^38^ and is therefore not associated with the observed karyosome defects.

### dKDM5 demethylase activity regulates meiotic transcription

**Figure 7:**
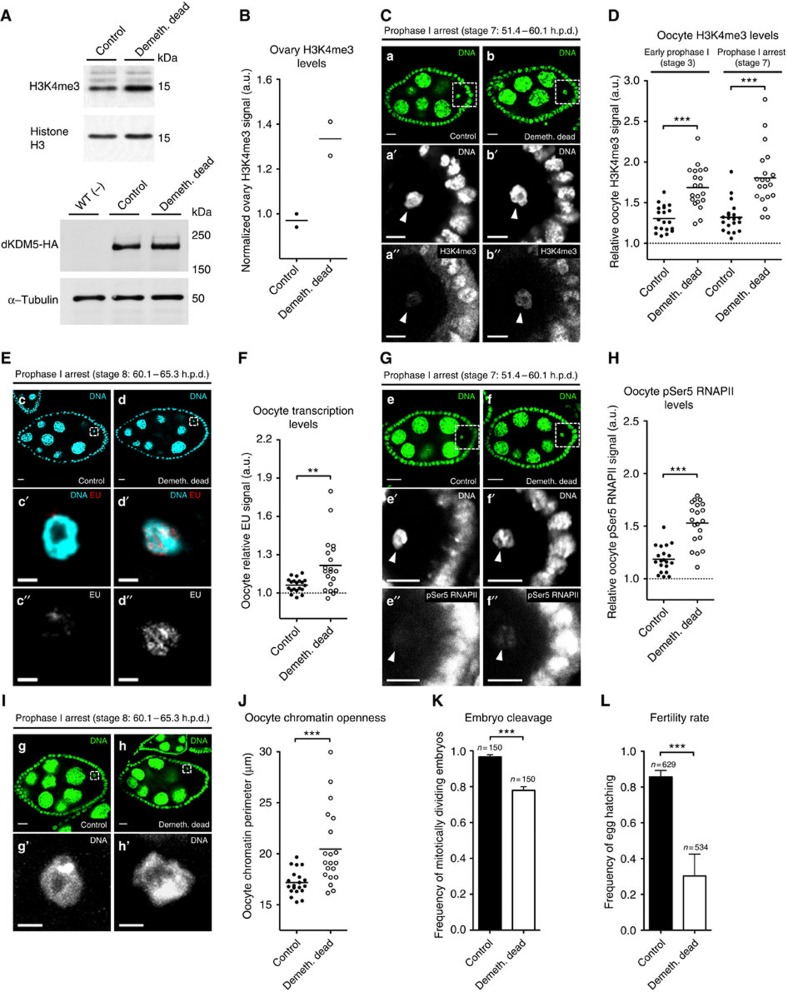
The demethylase activity of dKDM5 is required for oocyte transcriptional regulation and female fertility. (**A**,**B**) Loss of dKDM5 demethylase activity (demethylase-dead genomic dKDM5 transgene; Demeth. dead) significantly increases histone H3 lysine 4 trimethylation (H3K4me3) levels in the ovary. The normalized ratio between the H3K4me3 and histone H3 signals is expressed in arbitrary units (a.u.; see **B**). Protein immunoblots for dKDM5-HA (bottom panels) confirm that the demethylase-dead allele does not impair dKDM5 protein stability (quantification in Supplementary Fig. 12). WT (−) corresponds to flies without the HA-tagged transgenes. (**C**,**D**) Loss of dKDM5 demethylase activity significantly increases oocyte H3K4me3 (**a**–**b′′**). Oocyte H3K4me3 levels were compared before (oogenesis stage 3) and after (stage 7) the establishment of the prophase I arrest (Mann–Whitney *U*-test; *P*<0.0001). (**E**,**F**) Loss of dKDM5 demethylase activity induces premature oocyte transcription reactivation during the prophase I arrest (**c**–**d′′**) Oocyte transcription was measured by incorporation of the ethynyl uridine (EU) nucleotide (Mann–Whitney *U*-test; *P*=0.0084). (**G**,**H**) Loss of dKDM5 demethylase activity increases the levels of Ser5-phosphorylated RNA polymerase II in oocyte chromatin (pSer5 RNAPII; **e**–**f′′**; Mann–Whitney *U*-test; *P*<0.0001). (**I**,**J**) Precocious prophase I oocyte chromatin remodeling after loss of dKDM5 demethylase activity (**g**–**h′**). Oocyte chromatin openness was measured by calculating the perimeter of total chromatin volume (see **J**; Mann–Whitney *U*-test; *P*=0.0004). (**K**) Loss of dKDM5 demethylase activity impairs the initiation of embryonic mitotic divisions after fertilization. Error bars represent standard deviation (Student's *t*-test; *P*=0.0002). (**L**) Loss of dKDM5 demethylase activity significantly reduces *Drosophila* female fertility. Error bars represent s.d. (Student's *t*-test; *P*=0.0001). (**B**–**L**) Development time in relation to the start of oogenesis is expressed in hours post-germ line stem cell division (h.p.d.). Rectangles delimit the area of the depicted oocyte insets and arrowheads point to the oocyte's chromatin. Scale bars, 10 μm for ovarian follicles, 5 μm (**a′**–**b′′** and **e′**–**f′′**) and 2 μm (**c′**–**d′′** and **g′**–**h′**) for oocyte insets. Results of each independent experiment are plotted, horizontal lines specify mean values and asterisks indicate significant difference. Please see Supplementary Fig. 4 to compare phenotypical penetrance against a transheterozygous *dkdm5*^*−/−*^ mutant.

To understand to which extent the observed phenotypes were dependent on the demethylase activity of dKDM5, we engineered transgenic flies carrying two genomic copies of either wild type or a demethylase-dead dKDM5 variant (the previously described JmjC* mutation^22^) in a *dkdm5*^*−/−*^ mutant background. This demethylase-dead variant resulted in an increase of oocyte H3K4me3 levels comparable to that of dKDM5-depleted conditions (Fig. 7A–D).

Significantly, we observed that loss of dKDM5 demethylase activity resulted in defects similar to those recorded after dKDM5 depletion: premature oocyte transcription reactivation (Fig. 7E,F), increased oocyte pSer5 RNAPII levels (Fig. 7G,H), precocious oocyte chromatin remodelling (Fig. 7I,J), impaired entry into embryogenesis after fertilization (Fig. 7K), and reduced fertility (Fig. 7L). These data further validate the requirement of correct germ line H3K4me3 levels for normal oocyte gene expression regulation and chromatin remodelling. Since the penetrance of the aforementioned phenotypes was nevertheless lower than in dKDM5-depleted conditions (Supplementary Fig. 4), our observations using a demethylase-dead variant also suggest that other dKDM5-dependent epigenetic modifications are necessary for normal oogenesis.

In this regard, we failed to detect in the demethylase-dead background the decrease in oocyte H3K9ac seen under dKDM5-depleted conditions (Supplementary Fig. 8A,B). The discrepancy in oocyte H3K9ac levels between these two experimental conditions may be related to the previously described association of KDM5 with different chromatin-remodelling enzymes^27^,^28^. More specifically, we speculate that dKDM5 indirectly regulates H3K9ac and the extent of this regulation is less affected by a demethylase-dead mutation when compared to dKDM5-depleted conditions.

Altogether, our results strongly suggest that the oocyte needs to regulate its epigenome during early oogenesis (by restricting H3K4me3 levels, among other possible chromatin modifications) to temporally regulate its gene expression program and the remodelling of meiotic chromosomes in late prophase I. Our observations establish a new paradigm on the functions of the oocyte epigenome, whereby it serves as a chromatin-priming mechanism for the transcriptional regulation and remodelling of meiotic chromosomes.

## Discussion

**Figure 8:**
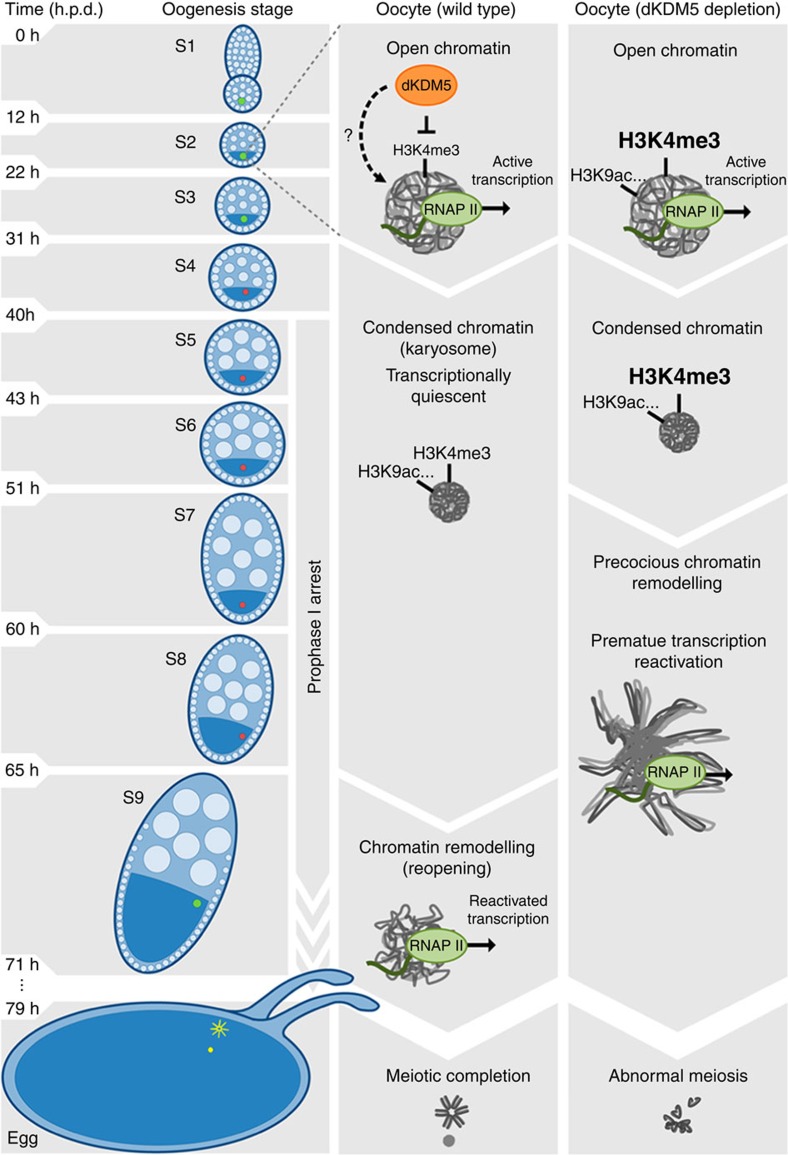
Proposed model for the epigenetic regulation of prophase I chromosome activity. The histone demethylase dKDM5 programs the oocyte chromatin state during early oogenesis, through its actions on H3K4me3 and other epigenetic modifications such as H3K9ac. Once programmed, the dKDM5-dependent oocyte epigenome temporally controls, several hours afterwards in late prophase I, the onset of transcription and meiotic chromosome remodeling. Ultimately, the germ line-specific activity of dKDM5 is required for successful completion of meiosis and female fertility. Development time in relation to the start of oogenesis is expressed in hours post-germ line stem cell division (h.p.d.).

Here we describe the unique diversity and dynamism of the *Drosophila* oocyte epigenome and provide evidence on how this epigenome can control chromosome activity during the prophase I arrest. According to our model (Fig. 8), dKDM5 sets the correct levels of H3K4me3 (and other epigenetic modifications such as H3K9ac) during early oogenesis, prior to the compaction of the meiotic chromosomes into the karyosome. Once specified, the dKDM5-dependent oocyte epigenome regulates, several hours later in late prophase I, two interconnected processes: transcription and chromosome remodelling. While the causal relationship between these two processes is still open to speculation, the developmentally regulated reactivation of transcription during the prophase I arrest is clearly dependent on the levels of RNAPII in oocyte chromatin. Even though the contribution of other epigenetic modifications to the phenotypes observed following dKDM5 depletion should be taken into consideration^27^,^39^, our data suggest that the oocyte epigenome is able to control RNAPII activity through an H3K4me3-mediated process. Therefore, we propose a temporally differentiated epigenetic mechanism where the removal of H3K4me3 marks in the open chromatin of early oogenesis regulates, several hours later, the onset of transcription in condensed chromatin.

Is transcriptional reactivation during the prophase I arrest functionally relevant, or is it merely a by-product of oocyte chromatin remodelling? As the identity of the karyosome-expressed genes is unknown, this question will remain unanswered. Nevertheless, the possibility of late prophase I gene expression being required for meiotic progression is tantalizing. In this regard, recent studies have shown that transcription in budding yeast shapes the meiotic chromosome axis^40^; while in human cells the late mitotic expression of a long non-coding RNA is required for the recruitment of centromeric proteins to the chromosomes^41^,^42^. We show that oocyte transcriptional reactivation coincides with a significant remodelling of the karyosome-clustered chromosomes prior to meiotic resumption, and that such remodelling is abnormally long and structurally different in dKDM5-depleted conditions. This remodelling of chromosomes in late prophase I is an evolutionarily conserved process required for the acquisition of meiotic maturation competence^43^,^44^. Therefore, the severely compromised meiotic completion observed following dKDM5 knockdown most likely results from the aforementioned defects in late prophase I chromosome remodelling.

To what extent can our findings be extended to mammalian oogenesis? Similar to *Drosophila*, mammalian oocytes have a diversified epigenome with a simultaneous enrichment of euchromatic and heterochromatic marks^17^,^45^. In addition, mammalian oocytes also change their transcriptional activity during the prophase I arrest^46^,^47^,^48^. In light of this, we propose that the significantly reduced female fertility of mouse mutants for KDM5B (one of the mammalian homologues of dKDM5 (ref. 49) is attributable to defects equivalent to those we report here.

In addition, the importance of this histone demethylase in fertility may even extend to humans. For several decades we and others have observed that deletions of the AZFb (AZoospermia Factor b) region of the human Y chromosome are causative agents of male infertility due to a complete block of sperm meiotic maturation^50^,^51^,^52^. The 6.2 Mb-long AZFb deletions can affect up to 10 different genes and, therefore, the molecular mechanism(s) responsible for the infertility phenotype remain(s) unknown^53^. One of the genes removed in AZFb deletions is KDM5D, the Y-chromosome-specific homologue of dKDM5 (ref. 54). Given the meiotic maturation defects seen in dKDM5-depleted oocytes, we speculate that KDM5D may be the long sought-after gene responsible for the AZFb deletion phenotype in humans.

## Methods

### *Drosophila* crosses

*Drosophila melanogaster* flies were raised using standard techniques. All *Drosophila* crosses were performed at 25 °C in polypropylene vials (51 mm diameter) containing enriched medium (cornmeal, molasses, yeast, soya flour and beetroot syrup). Approximately 20 virgin females were mated with 10 males and after 48 h flies were transferred to fresh vials. Twenty-four hours before analysis, female progeny (10 flies collected 3–7 days post eclosion) and five wild-type males (Oregon-R) were moved to polystyrene vials (23.5 mm diameter) containing standard medium (cornmeal, molasses, yeast and sucrose) supplemented with fresh yeast paste.

### Germ line-specific RNAi

The germ line-optimized Gal4-UAS (upstream activating sequence) system was used to express UAS-RNAi transgenes specifically in the female germ line^55^,^56^,^57^. UAS-RNAi males were crossed to either *nos-*GAL4 or UAS-Dcr2; *nos*-GAL4 virgin females^58^ to ensure potent germ line-specific gene knockdown during oogenesis. The *nanos* (*nos*) regulatory sequences ensure high levels of GAL4 expression from early oogenesis (germ line stem cell) to the mature egg^56^. For the list of RNAi lines used in the screen for regulators of the oocyte epigenome please consult Supplementary Table 1. It should be noted that the phenotypes measured in this screen were dependent not only on the biological activity of the tested regulators but also on the efficiency of the selected RNAi reagents. For example, such limitation may partially underlie the lack of a detectable effect on H3K4me3 following the depletion of the dKDM2 histone demethylase.

Two additional UAS-RNAi lines against dKDM5 were tested (Bloomington stock nos 35706 and 36652) and following concordant results stock 35706 was used for all reported experiments. As control for the RNAi experiments we used the *nos*-GAL4-driven expression of a double-stranded RNA against the mCherry fluorophore (the sequence for which is not present in the *Drosophila* genome; Bloomington stock no. 35785). For all germ line-specific knockdowns and corresponding controls at least 10 ovary pairs were analysed per genotype per experiment.

### Additional *Drosophila* strains

The two homozygous lethal alleles *dkdm5*^*10424*^ and *dkdm5*^*K06801*^ (Bloomington stock nos 12367 and 10403) were used to generate the *dkdm5*^*−/−*^ mutant (a *dkdm5*^*10424*^/*dkdm5*^*K06801*^ transheterozygote)^19^. Ovary dKDM5 levels were reduced by 80–90% in the *dkdm5*^*−/−*^ mutant (Supplementary Fig. 3) and adults eclose at a significantly lower frequency than expected. *dkdm5*^*−/−*^ mutant females display limited egg-laying capability despite still retaining morphologically normal oogenesis. For controls, a genomic rescue *dkdm5* transgene containing a C-terminal human influenza hemagglutinin (HA) tag was crossed into the *dkdm5*^*−/−*^ mutant background.

The genomic dKDM5 transgenes were generated by PCR using DNA from the strain *w*^*1118*^ and cloned into the vector pattB as an EcoRI/XhoI fragment. The demethylase-dead version of dKDM5 contains two point mutations that alter His637 and Glu639 to Alanine (JmjC* mutation^22^). To generate this transgene, the following primers were used: JmjC_F: 5′-CGCAGCCTTCTGCTGGGCCAACGCGGACCACTGGAGTA-3′; JmjC_R: 5′-TACTCCAGTGGTCCGCGTTGGCCCAGCAGAAGGCTGCG-3′. For controls, an 11.4 kb wild-type dKDM5 genomic rescue transgene was generated. This transgene encompasses all known dKDM5 transcripts. To generate this transgene, the following primers were used: pBGR_F1: 5′-CTAAAGGGAACAAAAGCTGGCGCAGTGCGACGGCTCCAAATAC-3′; pBGR_R1: 5′-CGTGTTGTCCGCCTCAGTTTTGGCGGACATAGCTTTAAGA-3′; pBGR_F2: 5′-CATCTTAAAGCTATGTCCGCCAAAACTGAGGCGGACAACA-3′; pGR_R2: 5′-CCCCGGGCTGCAGGAATTAACTCGGACTTGCACAAGCAGAAC-3′; pBGR_F3: 5′-CTAAAGGGAACAAAAGCTGGCGGCGGAGATTGTGGCCAGCTT-3′ and pBGR_R3: 5′-CCCCGGGCTGCAGGAATTGCTTACGCCGATTGCATGTC-3′. Both transgenes contain a C-terminal 3xHA-tag and were integrated into the attP site at 86Fb (Bloomington stock no. 24749; BestGene Inc.) under the control of the dKDM5 endogenous promoter. Transgenes were subsequently crossed into the *dkdm5*^*−/−*^ mutant background. The demethylase-dead mutant allele does not impair dKDM5 protein stability (Fig. 7A and Supplementary Fig. 12).

Mutant clones of follicle cells homozygous for the *dkdm5*^*10424*^ allele were generated following induction of the ‘FLP/FRT' recombination system^59^. Briefly, hs-FLP^22^; FRT40A, ubi-GFP males were crossed to w; *dkdm5*^*10424*^, FRT40/CyO females. Clones were induced by heat shocking third instar larvae for 1 h at 37 °C. *dkdm5*^*10424*^ follicle cell clones (somatic) were easily identifiable by the absence of an ubiquitous GFP signal.

### Antibodies

The oocyte epigenome was defined by immunofluorescence staining of adult ovaries using a panel of 21 primary antibodies (Supplementary Table 2). In addition, the following primary antibodies were used: rabbit anti-H3K4me3 (1:1000 dilution, Active Motif 39160); rabbit anti-H3K27me3 (1:250 dilution, Active Motif 39158); rabbit anti-H3K9me2 (1:200 dilution, Upstate 07–441); rabbit anti-pSer2 RNAPII (1:500 dilution, Abcam ab5095); rabbit anti-pSer5 RNAPII (1:1000 dilution, Abcam ab5131); mouse anti-HA (1:100 dilution, Covance MMS-101P); rabbit anti-HA (1:500 dilution, Abcam ab9110) and mouse anti-Orb (clones 4H8 and 6H4, 1:30 dilution each, Developmental Studies Hybridoma Bank). The following primary antibodies were used for ovary and embryo immunoblotting: rat anti-dKDM5 (1:5,000 dilution, from F. Azorín, Institute of Molecular Biology of Barcelona, Spain); rabbit anti-H3K4me3 (1:500 dilution, Active Motif 39160); rat anti-HA (1:500 dilution, Roche 11867423001); rabbit anti-H3 (1:8,000 dilution, Cell Signalling Technology #9715) and mouse anti-α-Tubulin (1:50,000 dilution, Sigma T6199).

Secondary detection was performed with Cy3, Cy5 and HRP-conjugated antibodies at 1:1,000 (immunofluorescence) and 1:4,000 (immunoblotting) dilutions (Jackson ImmunoResearch).

### Ovary immunofluorescence

Adult ovaries (10 ovary pairs per sample per experiment) were processed according to the standard procedures^60^,^61^. Briefly, ovaries were dissected in PBS and fixed for 20 min in a heptane (Fluka)-fixative mix at 3:1. The fixative consisted of 4% formaldehyde (Polysciences) in a PBS+0.5% NP-40 (Sigma) solution. Following gentle partial detachment of the ovarioles, ovaries were incubated for 2 h in PBST (0.2% Tween 20 (Sigma) in PBS) supplemented with 1% Triton X-100 (Sigma), 1% (w/v) bovine serum albumin (BSA; Sigma) and 1% (w/v) donkey serum (Sigma). Primary antibodies incubation was performed overnight at 4 °C in PBST supplemented with 1% BSA and 1% donkey serum (BBT solution). The following day, ovaries were incubated for 1 h at room temperature with the appropriate secondary antibodies diluted in BBT solution. Prior to mounting in fluorescence mounting medium (Dako) or VECTASHIELD (Vector Laboratories) without DAPI, DNA was stained for 30 min at room temperature using 1:5,000 SYTOX Green (Invitrogen) in PBST supplemented with 5 μg ml^−1^ RNase A (Sigma). Alternatively, VECTASHIELD supplemented with 1.5 μg ml^−1^ DAPI was used. Fluorescence images were acquired with a × 40 HCX PL APO CS oil immersion objective (numerical aperture: 1.25–0.75) and a × 63 HCX PL APO oil immersion objective (numerical aperture: 1.40–0.60) on a Leica SP5 confocal microscope.

### Oocyte transcription detection

The spatiotemporal detection of global oocyte RNA transcription was performed using a short-term ovary culture incorporation assay. Adult ovaries (10 ovary pairs per sample per experiment) were dissected in Grace's insect medium (Sigma) and transferred to a microcentrifuge tube containing fresh medium. Ovaries were incubated at room temperature for 30 min before 1 mM of the modified nucleotide ethynyl uridine (EU) was added to the medium. Following a 30-min incubation in the EU-supplemented medium, ovaries were fixed as previously described for immunofluorescence. The EU signal was processed according to the manufacturer's instructions (Click-iT RNA Alexa Fluor 594 Imaging Kit; ThermoFisher Scientific). Prior to mounting in fluorescence mounting medium (Dako), DNA was stained for 15 min at room temperature using Hoechst 33342 (ThermoFisher Scientific; 0.15 μg ml^−1^ in PBS). Fluorescent images were acquired with a × 40 HCX PL APO CS oil immersion objective (numerical aperture: 1.3) on a Leica SP5 confocal microscope.

The specificity of this assay for nascent RNA was tested by culturing ovaries in Grace's insect medium supplemented with EU and 5 μg ml^−1^ of the transcriptional inhibitor Actinomycin D (Sigma; Supplementary Fig. 13).

### Signal analysis and quantification

Oogenesis stages and their duration were defined according to the standard morphological features of the ovarian follicles and their frequency distribution in an ovariole sample^9^,^62^,^63^. For signal quantification, the entire chromatin volume of individual oocytes was acquired as 1 μm-thick slices using a Leica SP5 confocal microscope. Slices corresponding to individual oocytes were stacked into a single maximum intensity Z-projection plane using ImageJ software (v1.48i; National Institutes of Health). An outline was drawn around each oocyte's chromatin and the mean fluorescence value of the tested signals was recorded in arbitrary units (a.u.) alongside that of a background reading. For each oocyte, relative signals were calculated as the mean fluorescence of the oocyte signal divided by the mean fluorescence of its corresponding background. A minimum of three independent experiments were conducted for each experimental condition. For the calculation of relative histone PTM expression levels in the ovarian follicle cell types (at stage 4 of oogenesis), histone PTM signals were calculated per number of pixels in the nucleus and normalized to the corresponding DNA signal. To compare between the three cell types, the levels of each histone PTM were divided by the highest expression level recorded across all cell types. A total of three replicates were analysed for each data point. H3K4me3 signal quantification in somatic cells from stage 4 ovarian follicles was based on the ratio between the mean fluorescence of the tested cell divided by the mean fluorescence of an adjacent control cell (GFP positive).

### Chromatin perimeter analysis

The entire chromatin volume of individual oocytes and polar bodies was acquired as 0.2 μm-thick slices using a Leica SP5 confocal microscope. Slices were stacked into maximum intensity Z-projections and binarized using an automated global thresholding method (Huang's fuzzy thresholding; ImageJ). On the resulting image, the perimeter of the binarized chromatin signal was measured. A minimum of three independent experiments was conducted for each experimental condition.

### Meiotic metaphase I arrest analysis

We employed a recent protocol optimized for the analysis of the true secondary meiotic arrest configuration in *Drosophila* eggs^64^. Under these optimized, non-activated conditions, the *Drosophila* metaphase I arrest has been shown to correspond to a tightly packaged chromosome mass. Briefly, virgin females were collected and aged, in the absence of males, for 4 days in standard medium supplemented with fresh yeast paste. Ovaries (10 pairs per sample per experiment) were quickly dissected in modified Robb's medium^63^ and immediately transferred to fixative (4% formaldehyde in Robb's medium). Following a 5 min incubation, ovaries were processed as previously described for immunofluorescence and DNA was stained for 30 min at room temperature using 1:5,000 SYTOX Green (Invitrogen) in PBST supplemented with 5 μg ml^−1^ RNase A (Sigma). Fluorescence images were acquired with a × 63 HCX PL APO oil immersion objective (numerical aperture: 1.40–0.60) on a Leica SP5 confocal microscope. A minimum of two independent experiments was conducted for each experimental condition.

### Protein immunoblotting

Two types of ovary protein extracts were prepared: one for total protein extraction, the other for core histone purification. Starting material consisted of 10 ovary pairs per sample for total protein extraction and 20 pairs for core histone purification. For total protein extraction, ovaries were homogenized in a buffer containing 50 mM Tris-HCl (pH 7.5), 150 mM NaCl, 2 mM EDTA, 0.1% NP-40, 2 mM DTT, 10 mM NaF and protease inhibitor (Roche). For core histone purification (including their post-translational modifications), ovaries were processed using a histone purification mini kit (Active Motif).

Embryo total protein extracts were prepared from transcriptionally silent embryos (those prior to zygotic genome activation). Embryos (maximum 1 h post-egg laying) were dechorionated with a 50% commercial bleach solution and manually selected for the lack of observable pole cells and cortical nuclei (morphological hallmarks of a pre-ZGA state). Fifteen embryos were selected per genotype per experiment. Protein extracts were obtained by lysing the embryos with a needle in Laemmli sample buffer and heating for 5 min at 100 °C (ref. 65).

Protein samples were run on 6% or 14% SDS–polyacrylamide gel electrophoresis gels (for dKDM5 and H3K4me3 analysis, respectively) and proteins were transferred to Hybond-ECL membranes (Amersham). Membranes were blocked overnight at 4 °C in 5% non-fat milk in PBT followed by primary antibody incubation (overnight incubation at 4 °C). Secondary antibodies were added after washing with PBT and membranes were incubated for 2 h at room temperature. Protein detection was performed using ECL solution for 1 min and Hyperfilm ECL (Amersham). A minimum of two independent experiments were conducted for each experimental condition. Uncropped images of all protein blots can be found in Supplementary Fig. 14.

### *Drosophila* early embryo analyses

For polar body analysis, embryos were collected up to 30 min after egg laying. For embryo cleavage analysis, embryos were collected up to 1 h after egg laying and further incubated for 2 h at 25 °C. Embryo DNA staining was performed according to the standard procedures^61^. Briefly, embryos were dechorionated with a 50% commercial bleach solution and fixed for 1 h in a 4:1 heptane:fixative (4% formaldehyde in PBS) mix. Devitellinization was achieved by vigorously shaking fixed embryos in methanol for 1 min. Following rehydration, embryos were incubated for 30 min at room temperature in a 1:5,000 SYTOX Green solution (in 5 μg ml^−1^ RNase A-supplemented PBST). Embryos were mounted in fluorescence mounting medium and images were acquired with a × 40 HCX PL APO CS oil immersion objective (numerical aperture: 1.25–0.75) on a Leica SP5 confocal microscope. Four independent experiments were conducted for each experimental condition.

### Female fertility test

Egg-laying cages with 20 females under testing and 10 wild-type males (Oregon-R) were set-up and maintained at 25 °C for 48 h prior to analysis. All flies were 3–7 days post eclosion and analyses were performed on two consecutive days. A timed egg-lay (30 min) was conducted using apple juice agar plates as substrate. The total number of eggs harvested per plate was determined and plates were incubated for 48 h at 25 °C. The number of hatched eggs was then recorded and the fertility rate determined as the number of hatched eggs divided by the number of harvested eggs. Four independent experiments were conducted for each experimental condition.

### Statistical analysis

Relative fluorescence signals and chromatin perimeter measurements were compared between control and experimental groups using nonparametric tests (Mann–Whitney *U-*test). Frequencies of egg hatching and mitotic entry were compared between control and experimental groups using Student's *t*-test for two samples. In the screen for oocyte chromatin remodelling enzymes, H3K4me3 and H3K27me3 levels were compared using Student's *t*-test for paired samples. The distribution of the different configurations of the MI arrest was compared between control and experimental groups using two-way analysis of variance. Reported *P* values correspond to two-tailed tests. All analyses were performed using Prism 5 software (GraphPad).

### Data availability

All relevant data and reagents are available from the authors.

## Additional information

**How to cite this article:** Navarro-Costa, P. *et al.* Early programming of the oocyte epigenome temporally controls late prophase I transcription and chromatin remodelling. *Nat. Commun.* 7:12331 doi: 10.1038/ncomms12331 (2016).

## Supplementary Material

Supplementary Information Figures 1-14, Supplementary Tables 1-2
