# Supplementary material for: Early programming of the oocyte epigenome temporally controls late prophase I transcription and chromatin remodelling
Source: Nat Commun. 2016 Aug 10;7:12331. doi: 10.1038/ncomms12331 (PMC4987523; doi:10.1038/ncomms12331)
Supplement: Supplementary Information — Figures 1-14, Supplementary Tables 1-2 [file ncomms12331-s1.pdf]

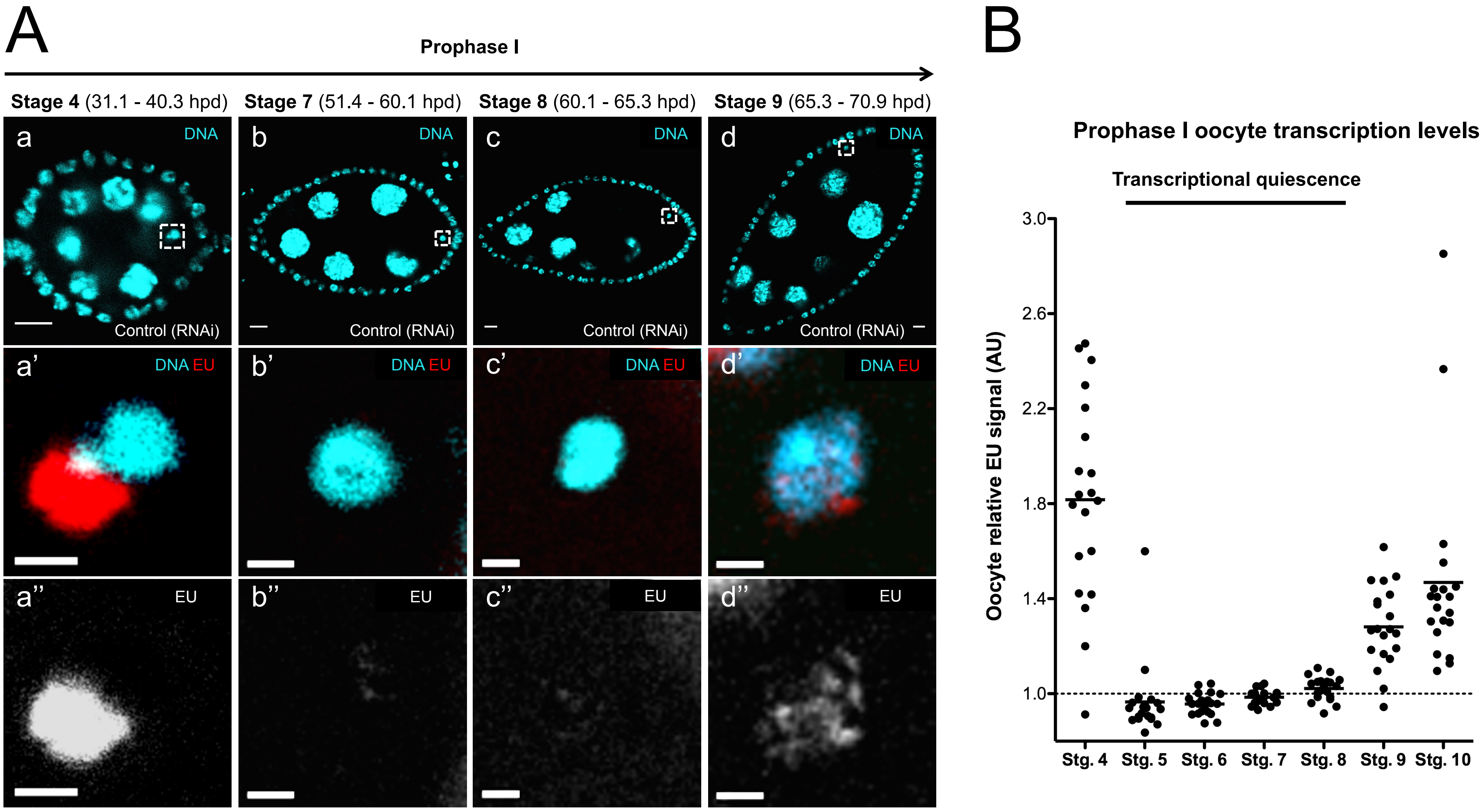
A
Prophase I
Stage 4 (31.1 - 40.3 hpd)
Stage 7 (51.4 - 60.1 hpd)
Stage 8 (60.1 - 65.3 hpd)
Stage 9 (65.3 - 70.9 hpd)
a
b
c
d
DNA
Control (RNAi)
a'
b'
c'
d'
DNA EU
a''
b''
c''
d''
EU
B
Prophase I oocyte transcription levels
Transcriptional quiescence
Oocyte relative EU signal (AU)
3.0
2.6
2.2
1.8
1.4
1.0
Stg. 4
Stg. 5
Stg. 6
Stg. 7
Stg. 8
Stg. 9
Stg. 10

Supplementary Figure 1. *Drosophila melanogaster* oocytes reactivate transcription in late prophase I.

A and **B**. *Drosophila melanogaster* oocytes reactivate transcription at oogenesis stage 9, after approximately 25h of transcriptional quiescence that starts with the onset of the prophase I arrest (stage 5; **a-d"**). Oocyte transcription levels were measured by incorporation of the modified nucleotide ethynyl uridine (EU). The specificity of this assay for nascent RNA was confirmed by treatment with the transcription inhibitor Actinomycin D (**Supplementary Fig. 13**). Signal quantification (see panel **B**) is expressed in fluorescence arbitrary units (AU). Horizontal lines specify mean values. Micrographs **b-d"** and the associated quantifications are also depicted in **Fig. 4**. Development time in relation to the start of oogenesis is expressed in hours post-germ line stem cell division (hpd). Rectangles delimit the area of the oocyte insets. Scale bars: 10 µm for ovarian follicles, 2 µm for oocyte insets.

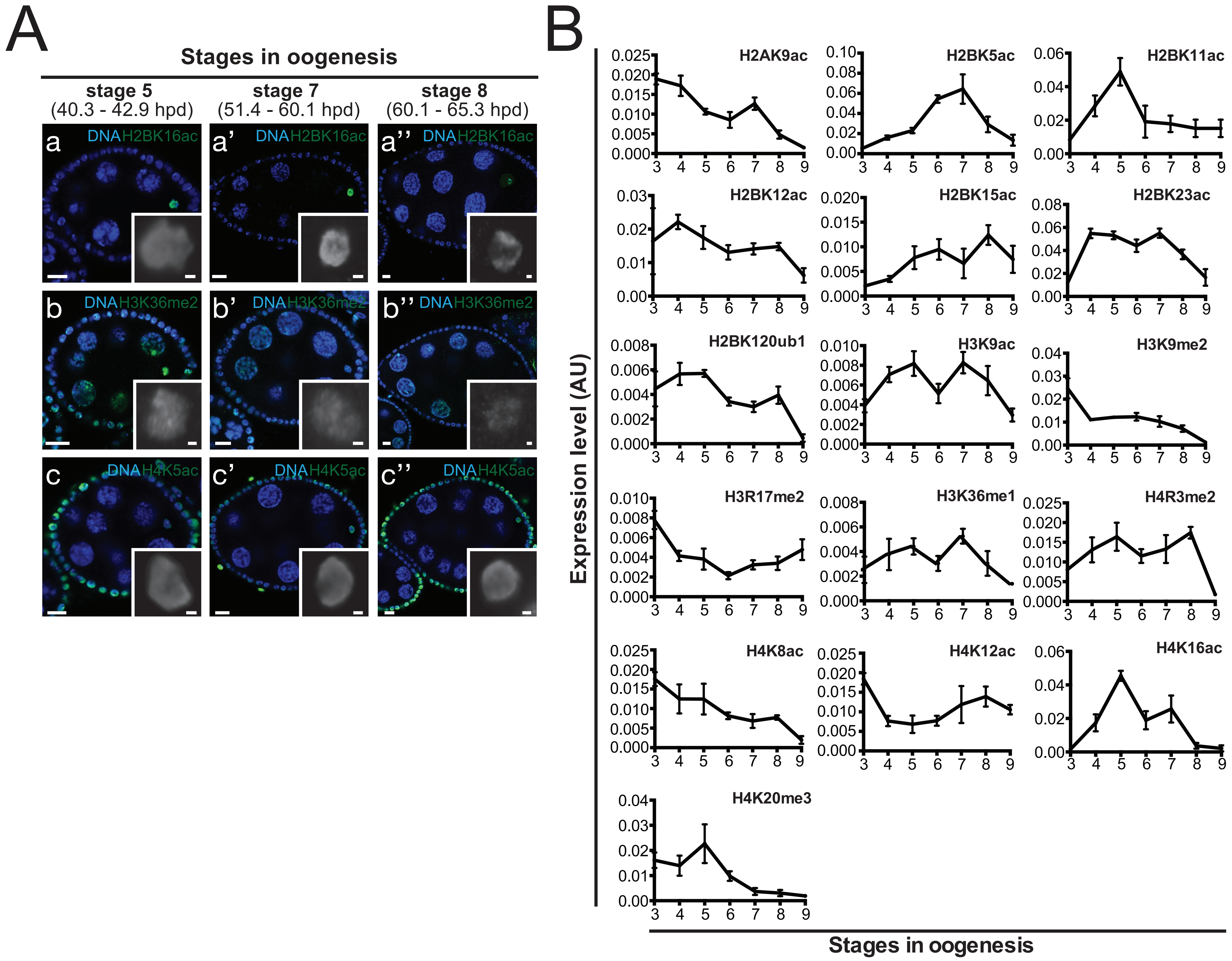
A
Stages in oogenesis
stage 5
(40.3 - 42.9 hpd)
stage 7
(51.4 - 60.1 hpd)
stage 8
(60.1 - 65.3 hpd)
a DNA H2BK16ac
a' DNA H2BK16ac
a'' DNA H2BK16ac
b DNA H3K36me2
b' DNA H3K36me2
b'' DNA H3K36me2
c DNA H4K5ac
c' DNA H4K5ac
c'' DNA H4K5ac
B
Expression level (AU)
H2AK9ac
H2BK5ac
H2BK11ac
H2BK12ac
H2BK15ac
H2BK23ac
H2BK120ub1
H3K9ac
H3K9me2
H3R17me2
H3K36me1
H4R3me2
H4K8ac
H4K12ac
H4K16ac
H4K20me3
Stages in oogenesis

Supplementary Figure 2. *Drosophila* oocytes have a dynamic epigenome.

A. Oocyte levels of histone H2B lysine 16 acetylation (H2BK16ac; **a-a''**), histone H3 lysine 36 dimethylation (H3K36me2; **b-b''**), and histone H4 lysine 5 acetylation (H4K5ac; **c-c''**) vary throughout prophase I. Insets depict oocyte chromatin histone post-translational modification (PTM) signals. Development time in relation to the start of oogenesis is expressed in hours post-germ line stem cell division (hpd). Scale bars: 10 µm for ovarian follicles and 1 µm for oocyte insets. See **Fig. 1D** for quantification. **B**. Temporal analysis of 16 histone PTMs in the oocyte chromatin from oogenesis stages 3 to 9 (approximately 49 hours). Relative expression levels are in fluorescence arbitrary units (AU) and were normalized to the corresponding DNA signal. Error bars represent standard deviation.

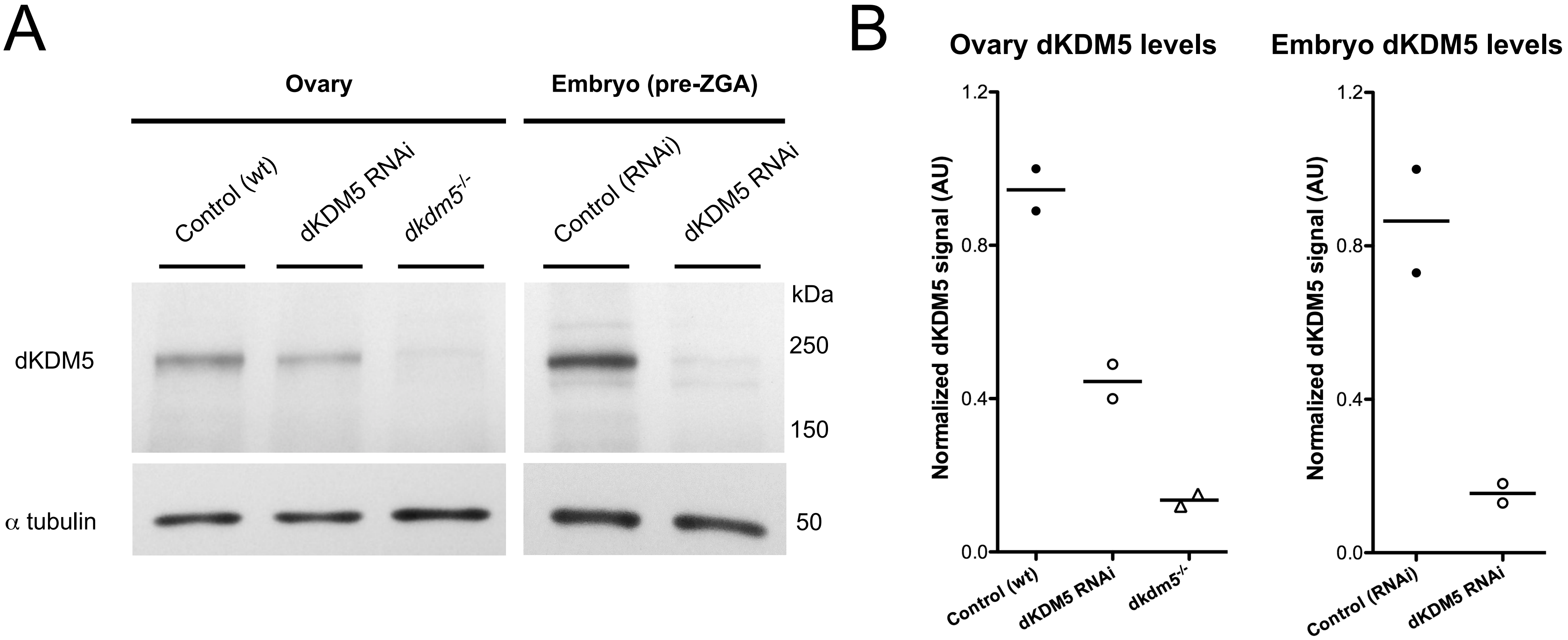
A
Ovary
Embryo (pre-ZGA)
Control (wt)
dKDM5 RNAi
dkdm5-/-
Control (RNAi)
dKDM5 RNAi
kDa
250
150
50
dKDM5
α tubulin
B
Ovary dKDM5 levels
Embryo dKDM5 levels
Normalized dKDM5 signal (AU)
1.2
0.8
0.4
0.0
Control (wt)
dKDM5 RNAi
dkdm5-/-
Control (RNAi)
dKDM5 RNAi

Supplementary Figure 3. Germ line dKDM5 levels are significantly reduced both under germ line-specific dKDM5 RNAi and in a *dkdm5*$^{-/-}$ mutant.

A and **B.** Both germ line-specific RNAi (*nos*-GAL4; UASp-dKDM5^{RNAi}) and a transheterozygous *dkdm5*$^{-/-}$ mutant (*dkdm5*^{10424}/*dkdm5*^{K06801}) displayed a significant depletion of dKDM5 in the female germ line. Protein immunoblots for dKDM5 were performed using extracts from whole ovaries (ovary) and manually-isolated embryos prior to the onset of zygotic genome activation [embryo (pre-ZGA)]. The ratio between the dKDM5 and α-tubulin signals were normalized and are expressed in arbitrary units (AU; see panel **B**). The results of each independent experiment are plotted and horizontal lines specify mean values. Control (wt) corresponds to Oregon-R flies.

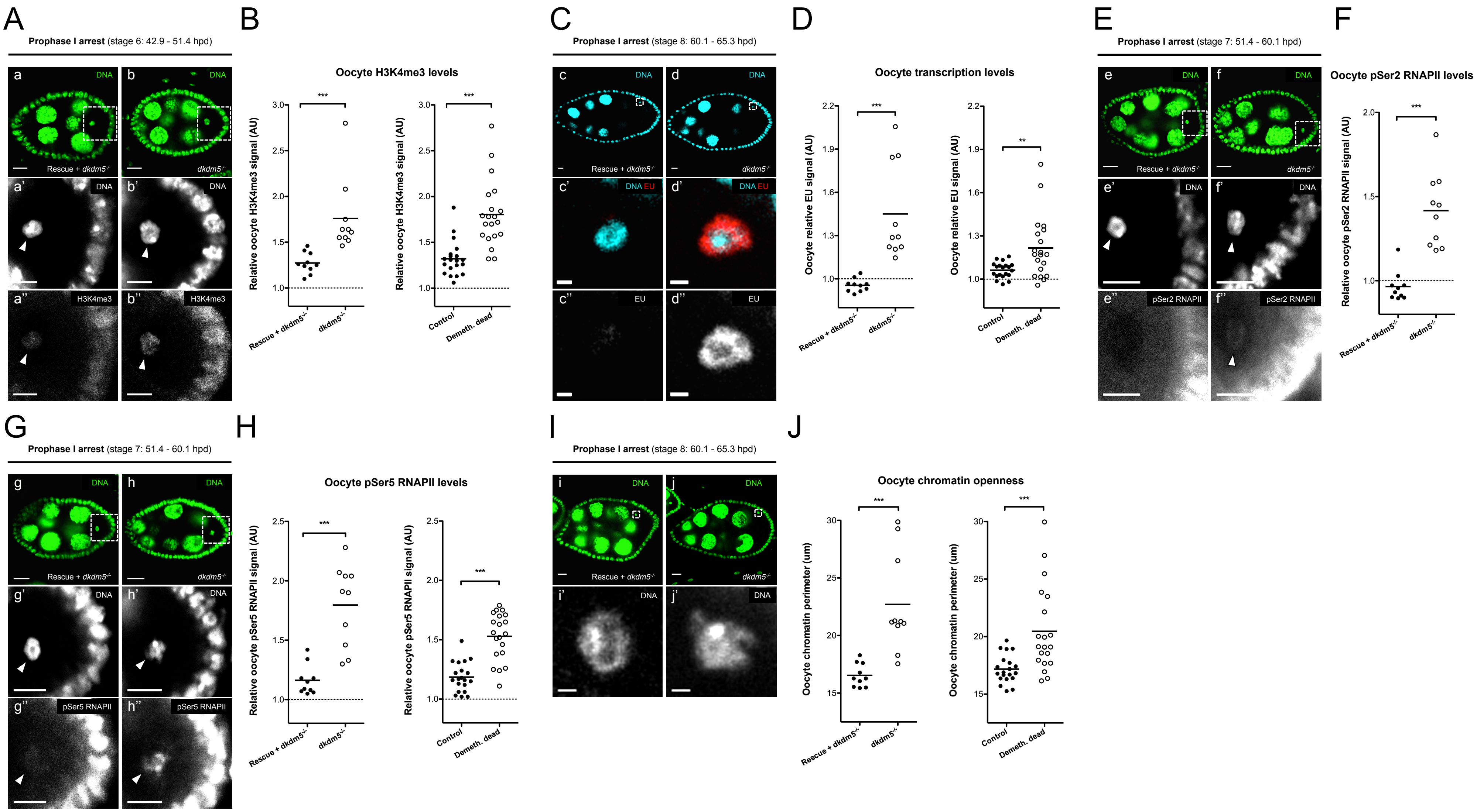
A
Prophase I arrest (stage 6: 42.9 - 51.4 hpd)
B
Oocyte H3K4me3 levels
Relative oocyte H3K4me3 signal (AU)
Rescue + dkdm5-/-
dkdm5-/-
Control
Demeth. dead
C
Prophase I arrest (stage 8: 60.1 - 65.3 hpd)
D
Oocyte transcription levels
Oocyte relative EU signal (AU)
E
Prophase I arrest (stage 7: 51.4 - 60.1 hpd)
F
Oocyte pSer2 RNAPII levels
Relative oocyte pSer2 RNAPII signal (AU)
G
Prophase I arrest (stage 7: 51.4 - 60.1 hpd)
H
Oocyte pSer5 RNAPII levels
Relative oocyte pSer5 RNAPII signal (AU)
I
Prophase I arrest (stage 8: 60.1 - 65.3 hpd)
J
Oocyte chromatin openness
Oocyte chromatin perimeter (um)

Supplementary Figure 4. A *dkdm5$^{-/-}$* mutant recapitulates the phenotypes observed under germ line-specific dKDM5 RNAi.

A transheterozygous *dkdm5$^{-/-}$* mutant (*dkdm5^{10424}/dkdm5^{K06801}*) displayed similar phenotypes to those recorded under germ line-specific RNAi (*nos*-GAL4; UASp-dKDM5^{RNAi}). For controls, a genomic rescue *dkdm5* transgene containing a C-terminal human influenza hemagglutinin (HA) tag was crossed into the *dkdm5$^{-/-}$* mutant background (Rescue + *dkdm5$^{-/-}$*). **A** and **B**. *dkdm5$^{-/-}$* oocytes have significantly increased levels of histone H3 lysine 4 trimethylation (H3K4me3; **a-b''**). Oocyte H3K4me3 levels were compared at stage 6 of oogenesis (representative the prophase I arrest). Signal quantification (see panel **B**) is expressed in fluorescence arbitrary units (AU) and compared to that of a demethylase-dead dKDM5 variant (**Fig. 7C,D**). Horizontal lines specify mean values and asterisks indicate significant difference (Mann-Whitney U test; P = 0.0002). A similar observation was recorded under germ line-specific RNAi (**Fig. 3A,B**). **C** and **D**. *dkdm5$^{-/-}$* oocytes prematurely reactivate transcription during the prophase I arrest (**c-d''**). Oocyte transcription was measured at oogenesis stage 8 by incorporation of the modified nucleotide ethynyl uridine (EU). Signal quantification (see panel **D**) is expressed in fluorescence AU and compared to that of a demethylase-dead dKDM5 variant (**Fig. 7E,F**). Horizontal lines specify mean values and asterisks indicate significant difference (Mann-Whitney U test; $P <$ 0.0001). A similar observation was recorded under germ line-specific RNAi (**Fig. 4A,B**). **E** and **F**. Significantly higher levels of transcriptional elongation were recorded in *dkdm5$^{-/-}$* oocytes (**e-f''**). Active RNA polymerase II

[phosphorylated at position serine 2 (pSer2) of the C-terminal repeat domain] levels were compared at stage 7 of oogenesis (representative of the prophase I arrest). Signal quantification (see panel **F**) is expressed in fluorescence AU. Horizontal lines indicate mean values and asterisks indicate significant difference (Mann-Whitney U test; P < 0.0001). A similar observation was recorded under germ line-specific RNAi (**Fig. 5C,D**). **G** and **H**. The chromatin of *dkdm5*$^{-/-}$ oocytes displays increased levels of RNA polymerase II phosphorylated at position serine 5 of the C-terminal repeat domain (pSer5 RNAPII; **g-h''**). pSer5 RNAPII levels were compared at stage 7 of oogenesis. Signal quantification (see panel **H**) is expressed in fluorescence AU and compared to that of a demethylase-dead dKDM5 variant (**Fig. 7G,H**). Horizontal lines indicate mean values and asterisks indicate significant difference (Mann-Whitney U test; P = 0.0006). A similar observation was recorded under germ line-specific RNAi (**Fig. 5A,B**). **I** and **J**. *dkdm5*$^{-/-}$ oocytes display precocious chromatin remodeling during the prophase I arrest (**i-j'**). Oocyte chromatin openness was measured by calculating the perimeter of total chromatin volume at stage 8 of oogenesis (representative the prophase I arrest) and is expressed in micrometres [µm; see panel **J** also for comparison with a demethylase-dead dKDM5 variant (**Fig. 7I,J**)]. Horizontal lines specify mean values and asterisks indicate significant difference (Mann-Whitney U test; P = 0.0006). A similar observation was recorded under germ line-specific RNAi (**Fig. 4C,D**). **A-J**. Development time in relation to the start of oogenesis is expressed in hours post-germ line stem cell division (hpd). Rectangles delimit the area of the depicted oocyte insets and arrowheads point to the

oocyte’s chromatin. Scale bars: 10 µm for ovarian follicles, 5 µm (**a'-b''** and **e'-h''**) and 2 µm (**c'-d''** and **i'-j'**) for oocyte insets.

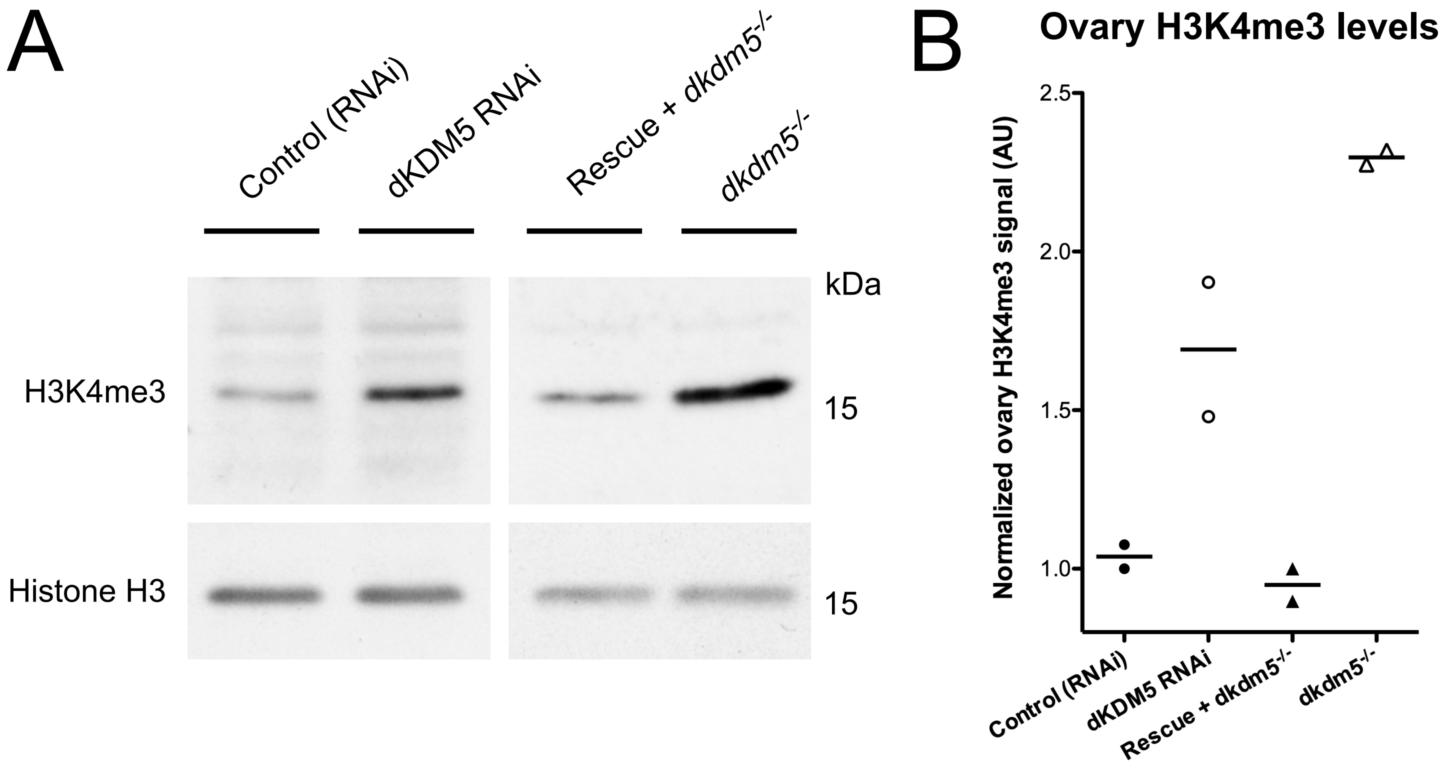
A
Control (RNAi)
dKDM5 RNAi
Rescue + dkdm5-/-
dkdm5-/-
kDa
H3K4me3
15
Histone H3
15
B
Ovary H3K4me3 levels
Normalized ovary H3K4me3 signal (AU)
2.5
2.0
1.5
1.0
Control (RNAi)
dKDM5 RNAi
Rescue + dkdm5-/-
dkdm5-/-

Supplementary Figure 5. H3K4me3 levels are significantly increased both under germ line-specific dKDM5 RNAi and in a *dkdm5*$^{-/-}$ mutant.

A and **B.** The levels of histone H3 lysine 4 trimethylation (H3K4me3) in the ovary are significantly increased upon knockdown of dKDM5 by germ line-specific RNAi (*nos*-GAL4; UASp-dKDM5^{RNAi}) or in a *dkdm5*$^{-/-}$ mutant (*dkdm5*^{10424}/*dkdm5*^{K06801}). Protein immunoblots for H3K4me3 were performed using histone extracts from whole ovaries. The ratio between the H3K4me3 and histone H3 signals were normalized and are expressed in arbitrary units (AU; see panel **B**). The results of each independent experiment are plotted and horizontal lines specify mean values. The control Rescue + *dkdm5*$^{-/-}$ corresponds to the crossing into the *dkdm5*$^{-/-}$ mutant background of a genomic rescue *dkdm5* transgene containing a C-terminal human influenza hemagglutinin (HA) tag.

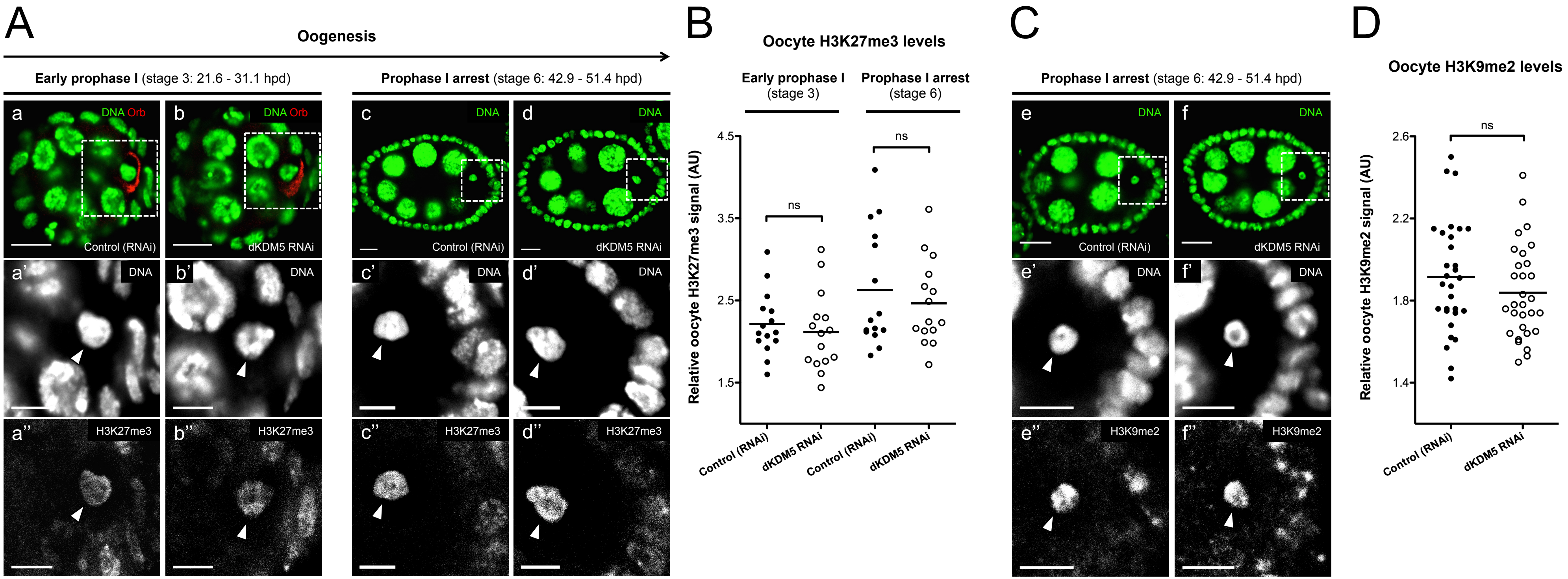
A
Oogenesis
Early prophase I (stage 3: 21.6 - 31.1 hpd)
Prophase I arrest (stage 6: 42.9 - 51.4 hpd)
a DNA Orb Control (RNAi)
b DNA Orb dKDM5 RNAi
c DNA Control (RNAi)
d DNA dKDM5 RNAi
a' DNA
b' DNA
c' DNA
d' DNA
a'' H3K27me3
b'' H3K27me3
c'' H3K27me3
d'' H3K27me3
B
Oocyte H3K27me3 levels
Early prophase I (stage 3)
Prophase I arrest (stage 6)
ns
ns
Relative oocyte H3K27me3 signal (AU)
4.5
3.5
2.5
1.5
Control (RNAi)
dKDM5 RNAi
Control (RNAi)
dKDM5 RNAi
C
Prophase I arrest (stage 6: 42.9 - 51.4 hpd)
e DNA Control (RNAi)
f DNA dKDM5 RNAi
e' DNA
f' DNA
e'' H3K9me2
f'' H3K9me2
D
Oocyte H3K9me2 levels
ns
Relative oocyte H3K9me2 signal (AU)
2.6
2.2
1.8
1.4
Control (RNAi)
dKDM5 RNAi

Supplementary Figure 6. Oocyte heterochromatin is not affected by the germ line-specific knockdown of the histone demethylase dKDM5.

A and **B**. Oocyte facultative heterochromatin (histone H3 lysine 27 trimethylation - H3K27me3) remains unchanged after the germ line-specific knockdown of dKDM5 (**a-d''**). Oocyte H3K27me3 levels were compared at stages 3 and 6 of oogenesis (representative of stages before and after the establishment of the prophase I arrest, respectively). **C** and **D**. Oocyte levels of constitutive heterochromatin (histone H3 lysine 9 dimethylation - H3K9me2) also remain unchanged after the germ line-specific depletion of dKDM5 (**e-f''**). **A-D**. Development time is expressed in hours post-germ line stem cell division (hpd). Rectangles delimit the area of the oocyte insets, arrowheads point to the oocyte's chromatin. In order to distinguish early prophase I oocytes from other germ line cells, an oocyte cytoplasm-specific staining was performed (against the RNA-binding protein Orb; **a**, **b**). Signal quantification (see panels **B** and **D**) is expressed in fluorescence arbitrary units (AU). Horizontal lines specify mean values and "ns" indicates no significant difference (Mann-Whitney U test). Scale bars: 10 µm for ovarian follicles, 5 µm for oocyte insets. Panel A micrographs **c** to **d''** are also depicted in **Fig. 3**.

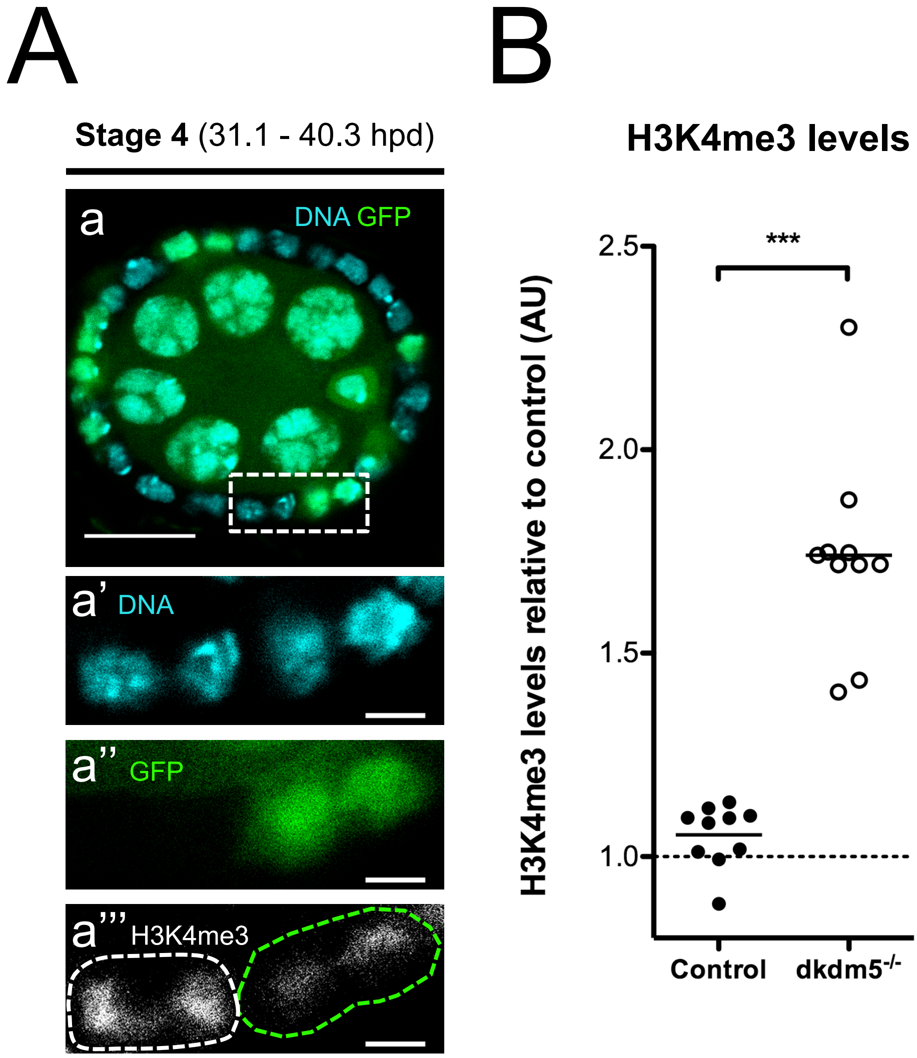
A
Stage 4 (31.1 - 40.3 hpd)
a
DNA GFP
a' DNA
a'' GFP
a''' H3K4me3
B
H3K4me3 levels
***
H3K4me3 levels relative to control (AU)
2.5
2.0
1.5
1.0
Control
dkdm5-/-

Supplementary Figure 7. *dkdm5*^{10424} mutant follicle cell clones display increased levels of H3K4me3.

A and **B**. Mutant clones of follicle cells homozygous for the *dkdm5*^{10424} allele (GFP negative) have significantly increased histone H3 lysine 4 trimethylation (H3K4me3) levels when compared with adjacent control cells (GFP positive; **a**-**a'''**). In **a'''**, the color-coded outlines delimit mutant (white) and control (green) cells. Signal quantification is expressed in fluorescence arbitrary units (AU; see panel **B**) and each measurement was normalized against an adjacent control cell. Horizontal lines specify mean values and asterisks indicate significant difference (Mann-Whitney U test; $P < 0.0001$). Scale bars: 10 µm for the ovarian follicle, 2 µm for follicle cell insets.

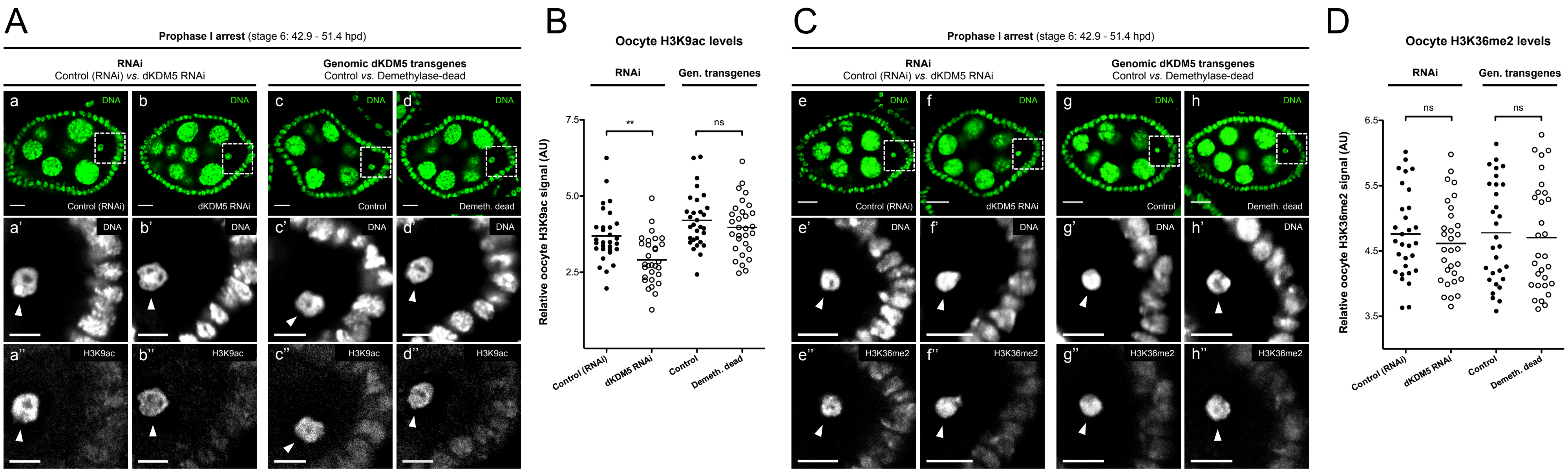
A
Prophase I arrest (stage 6: 42.9 - 51.4 hpd)
RNAi
Control (RNAi) vs. dKDM5 RNAi
Genomic dKDM5 transgenes
Control vs. Demethylase-dead
DNA
Control (RNAi)
dKDM5 RNAi
Control
Demeth. dead
H3K9ac
B
Oocyte H3K9ac levels
RNAi
Gen. transgenes
**
ns
Relative oocyte H3K9ac signal (AU)
7.5
5.0
2.5
Control (RNAi)
dKDM5 RNAi
Control
Demeth. dead
C
Prophase I arrest (stage 6: 42.9 - 51.4 hpd)
H3K36me2
D
Oocyte H3K36me2 levels
Relative oocyte H3K36me2 signal (AU)
6.5
5.5
4.5
3.5

Supplementary Figure 8. dKDM5 is required for normal levels of oocyte H3K9ac independently of its demethylase activity.

A and **B**. The levels of oocyte histone H3 lysine 9 acetylation (H3K9ac) were decreased after the germ line-specific knockdown of dKDM5 (**a-b''**), but remained unchanged after loss of dKDM5 demethylase activity (Demeth. dead; **c-d''**). **C** and **D**. Oocyte levels of histone H3 lysine 36 dimethylation (H3K36me2) remained unchanged after both the germ line-specific depletion of dKDM5 (**e-f''**) and after loss of dKDM5 demethylase activity (**g-h''**). Oocyte H3K9ac and H3K36me2 levels were compared at stage 6 of oogenesis (representative of the prophase I arrest). **A-D**. Development time is expressed in hours post-germ line stem cell division (hpd). Rectangles delimit the area of the oocyte insets, arrowheads point to the oocyte's chromatin. Signal quantification (see panels **B** and **D**) is expressed in fluorescence arbitrary units (AU). Horizontal lines specify mean values; asterisks indicate significant difference (Mann-Whitney U test; $P = 0.0012$) and "ns" indicates no significant difference. Scale bars: 10 µm for ovarian follicles, 5 µm for oocyte insets.

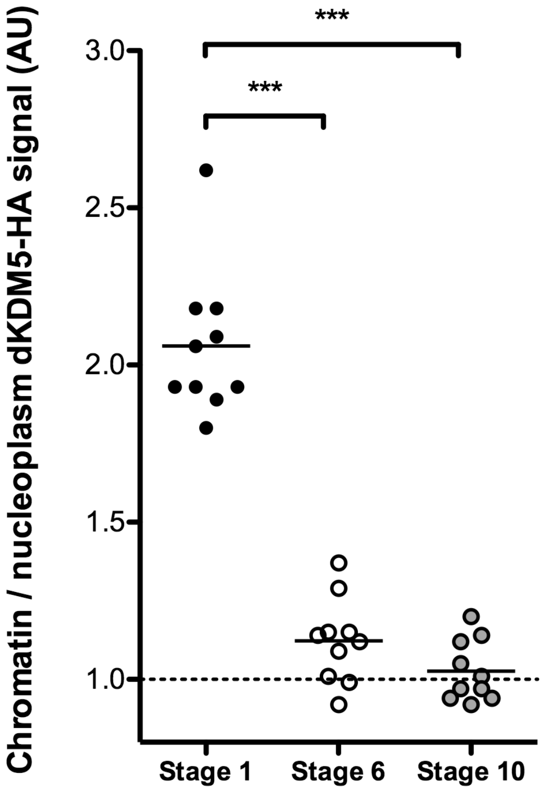
Chromatin / nucleoplasm dKDM5-HA signal (AU)
3.0
2.5
2.0
1.5
1.0
***
***
Stage 1
Stage 6
Stage 10

Supplementary Figure 9. dKDM5 is evicted from the oocyte's chromatin at the initial stages of oogenesis.

Oocyte chromatin compaction and transcriptional quiescence (oogenesis stage 6 as a representative example) were associated with a significant decrease of the dKDM5 signal localizing to the oocyte's chromatin. The low dKDM5 oocyte chromatin levels were maintained in late prophase I, even during the reactivation of oocyte transcription (stage 10 as a representative example). Oogenesis stage 1 corresponds to an early prophase I transcriptionally active oocyte. Oocyte chromatin signal quantification is expressed in fluorescence arbitrary units (AU) and each measurement was normalized with the corresponding nucleoplasmic signal. Horizontal lines specify mean values and asterisks indicate significant difference (Mann-Whitney U test; $P = 0.0002$). The dKDM5 signal corresponds to a genomic *dkdm5* transgene containing a C-terminal human influenza hemagglutinin (HA) tag crossed into the *dkdm5*$^{-/-}$ mutant background (antibody: anti-HA). Representative images of the dKDM5 signal across different stages of oogenesis are shown in **Fig. 3C**.

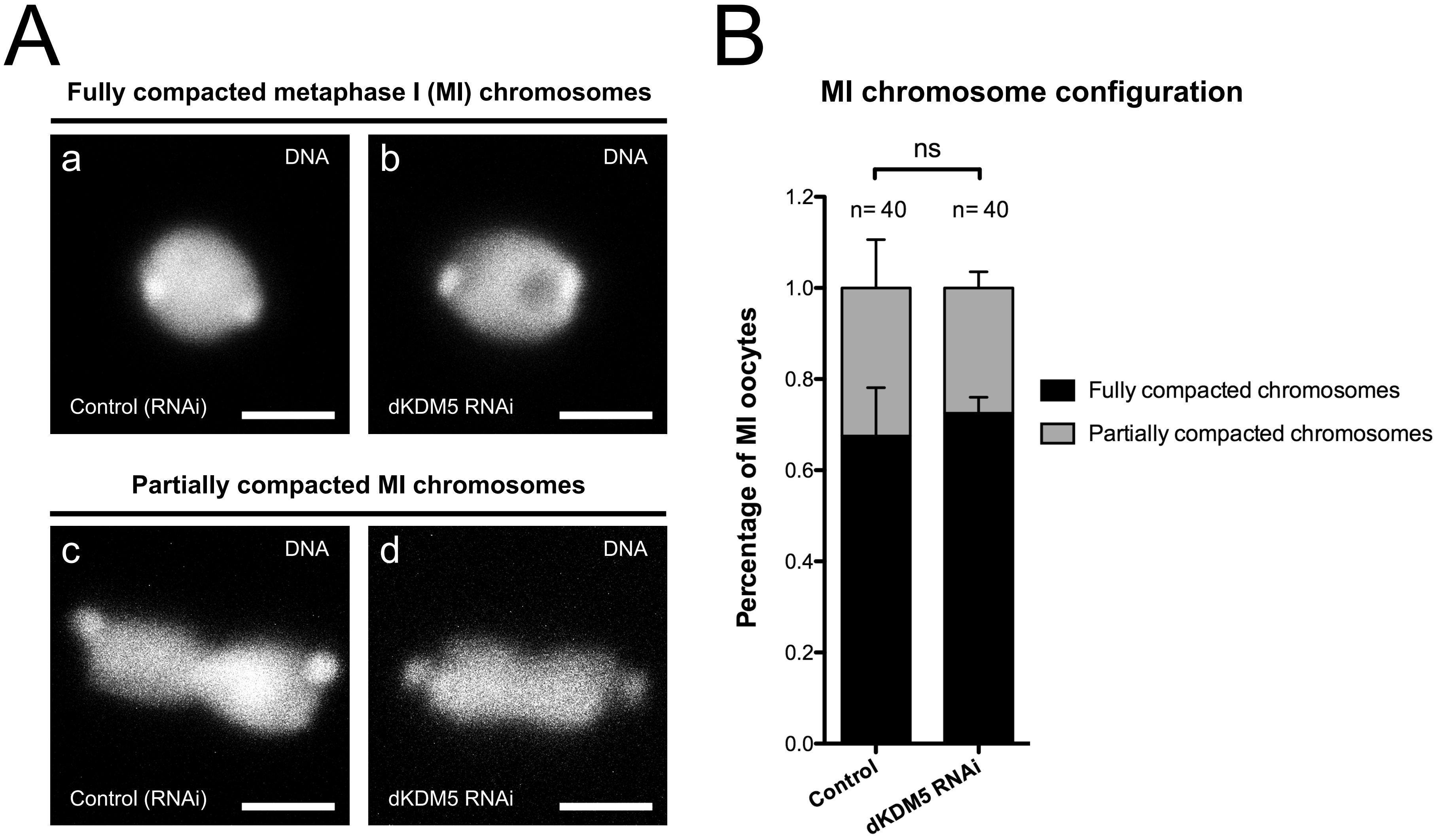
A
Fully compacted metaphase I (MI) chromosomes
a
DNA
Control (RNAi)
b
DNA
dKDM5 RNAi
Partially compacted MI chromosomes
c
DNA
Control (RNAi)
d
DNA
dKDM5 RNAi
B
MI chromosome configuration
ns
n= 40
n= 40
Percentage of MI oocytes
1.2
1.0
0.8
0.6
0.4
0.2
0.0
Control
dKDM5 RNAi
Fully compacted chromosomes
Partially compacted chromosomes

Supplementary Figure 10. The metaphase I (MI) arrest of mature oocytes is not visibly affected by the germ line-specific knockdown of the histone demethylase dKDM5.

A and **B**. Frequency and morphology of different configurations of the *Drosophila melanogaster* MI arrest were not noticeably altered in dKDM5-depleted conditions. Two different MI configurations were quantified: a tightly packaged chromosome mass (fully compacted chromosomes; **a**,**b**) and a more distended plate characterized by partial chromosome retraction (partially compacted chromosomes; **c**,**d**). The frequency of both configurations in mature, non-activated oocytes is represented in panel **B**. Error bars represent standard deviation and “ns” indicates no significant difference (Two-way ANOVA). Scale bars: 2 µm.

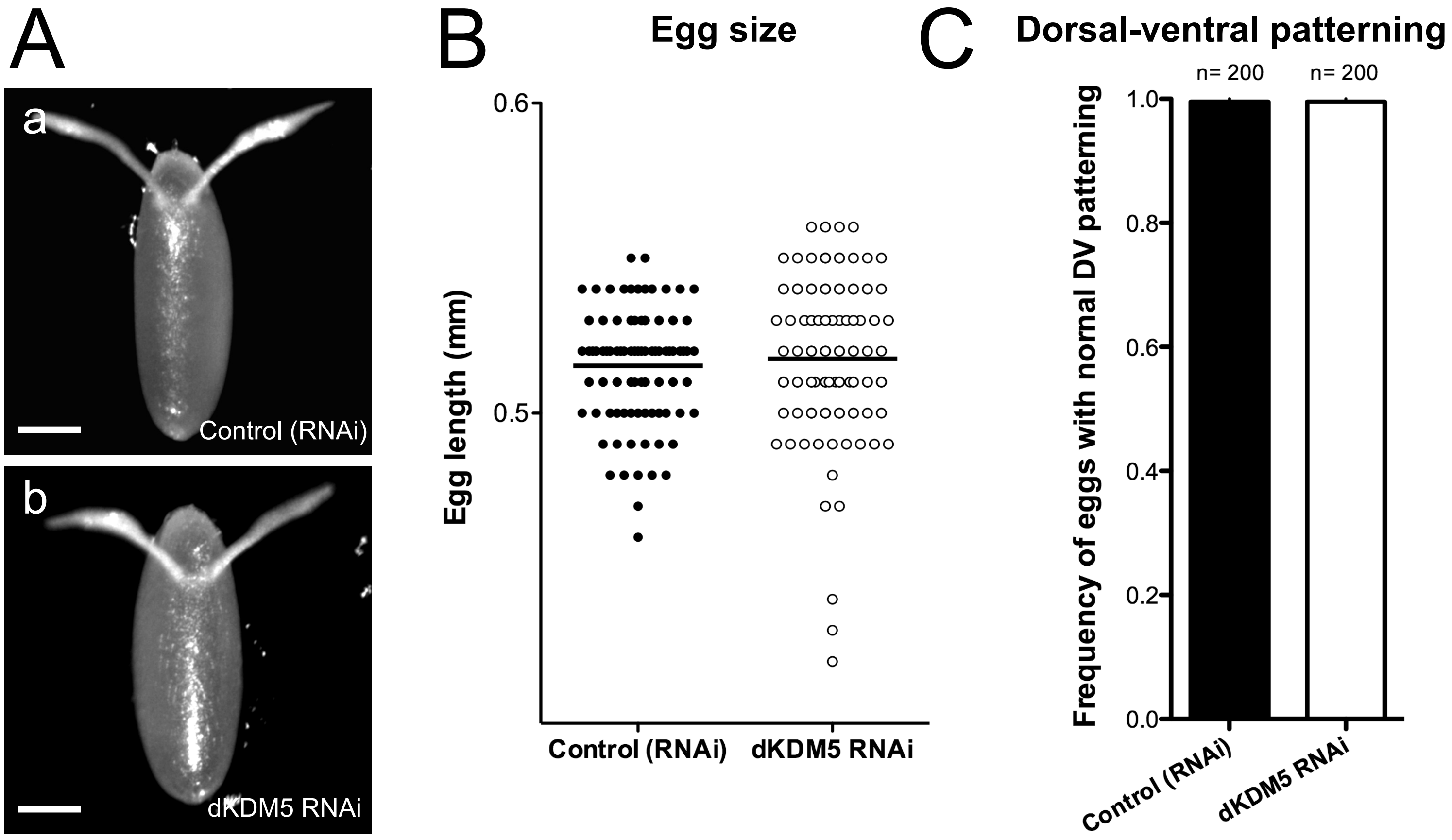
A
a
Control (RNAi)
b
dKDM5 RNAi
B
Egg size
Egg length (mm)
0.6
0.5
Control (RNAi)
dKDM5 RNAi
C
Dorsal-ventral patterning
n= 200
n= 200
Frequency of eggs with normal DV patterning
1.0
0.8
0.6
0.4
0.2
0.0
Control (RNAi)
dKDM5 RNAi

Supplementary Figure 11. The germ line-specific knockdown of the histone demethylase dKDM5 does not affect egg size or dorsal-ventral patterning.

A. Eggs collected from control [Control (RNAi)] and dKDM5-depleted conditions (dKDM5 RNAi) have similar size and morphology (**a** and **b**). Scale bars: 125 µm. **B**. Egg size is defined by the length of its main axis and is expressed in millimetres (mm). Horizontal lines specify mean values. **C**. Normal DV (dorsal-ventral) patterning was defined by the presence of two correctly spaced dorsal appendages of normal length. Error bars represent standard deviation.

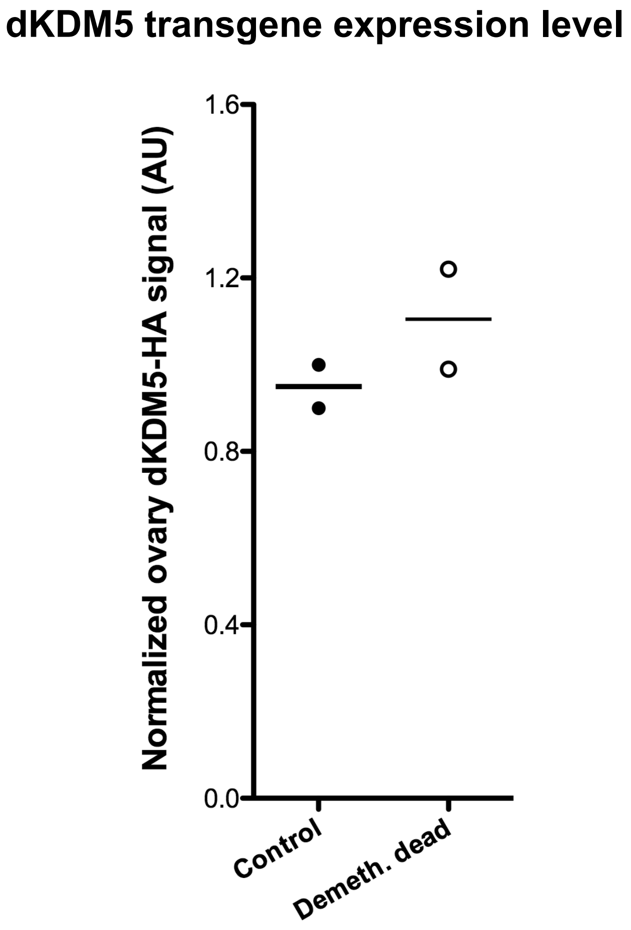

dKDM5 transgene expression level
Normalized ovary dKDM5-HA signal (AU)
1.6
1.2
0.8
0.4
0.0
Control
Demeth. dead

Supplementary Figure 12. The demethylase-dead mutant allele (JmjC* mutation) does not impair dKDM5 protein stability.

Transgenic dKDM5 protein levels in the demethylase-dead dKDM5 construct (Demeth. dead) are not decreased compared to control (wild type dKDM5 transgene). Protein immunoblots for C-terminal human influenza hemagglutinin (HA)-tagged dKDM5 transgenes were performed using whole ovaries extracts (antibody: anti-HA). The ratio between the dKDM5-HA and α-tubulin signals were normalized and are expressed in arbitrary units. The results of each independent experiment are plotted and horizontal lines specify mean values. Representative images of the protein immunoblots are shown in **Fig. 7A**.

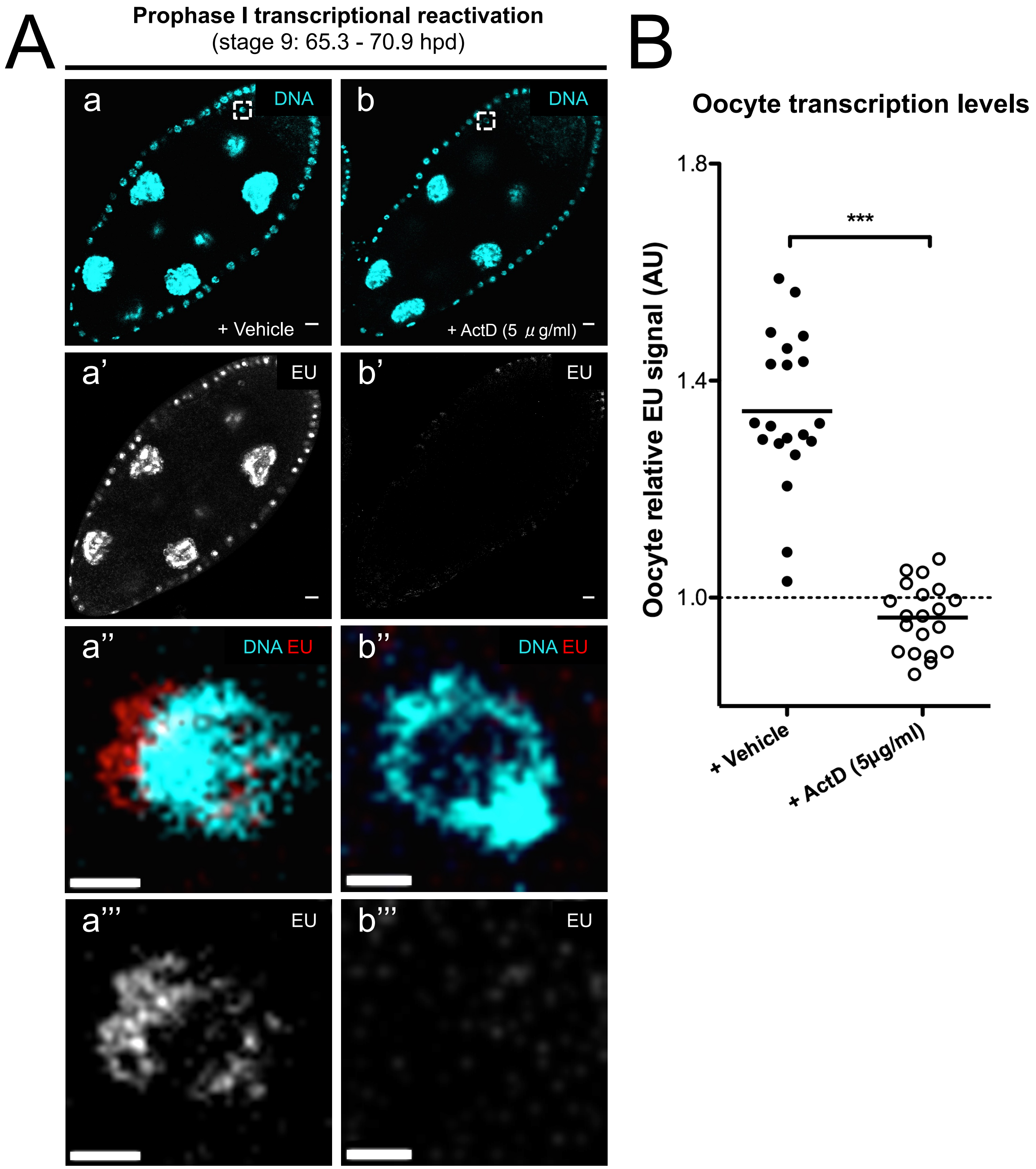
A
Prophase I transcriptional reactivation
(stage 9: 65.3 - 70.9 hpd)
a
DNA
+ Vehicle
b
DNA
+ ActD (5 µ g/ml)
a'
EU
b'
EU
a''
DNA EU
b''
DNA EU
a'''
EU
b'''
EU
B
Oocyte transcription levels
Oocyte relative EU signal (AU)
1.8
1.4
1.0
***
+ Vehicle
+ ActD (5µg/ml)

Supplementary Figure 13. Ethynyl uridine (EU) incorporation is abrogated by incubation with the transcriptional inhibitor Actinomycin D.

A and **B.** The specificity of the EU incorporation assay for nascent RNA was tested by supplementing ovary culture medium with 5 µg/ml Actinomycin D [+ ActD (5 µg/ml)] and measuring oocyte gene expression during prophase I transcriptional reactivation (oogenesis stage 9). Signal quantification (see panel **B**) is expressed in fluorescence arbitrary units (AU). Horizontal lines specify mean values and asterisks indicate significant difference (Mann-Whitney U test; $P < 0.0001$). Development time in relation to the start of oogenesis is expressed in hours post-germ line stem cell division (hpd). Rectangles delimit the area of the oocyte insets. The tested genotype corresponds to the RNAi control (*nos*-GAL4; UASp-mCherry^{RNAi}). Scale bars: 10 µm for ovarian follicles, 2 µm for oocyte insets.

Fig. 7; panel A (H3K4me3; H3)

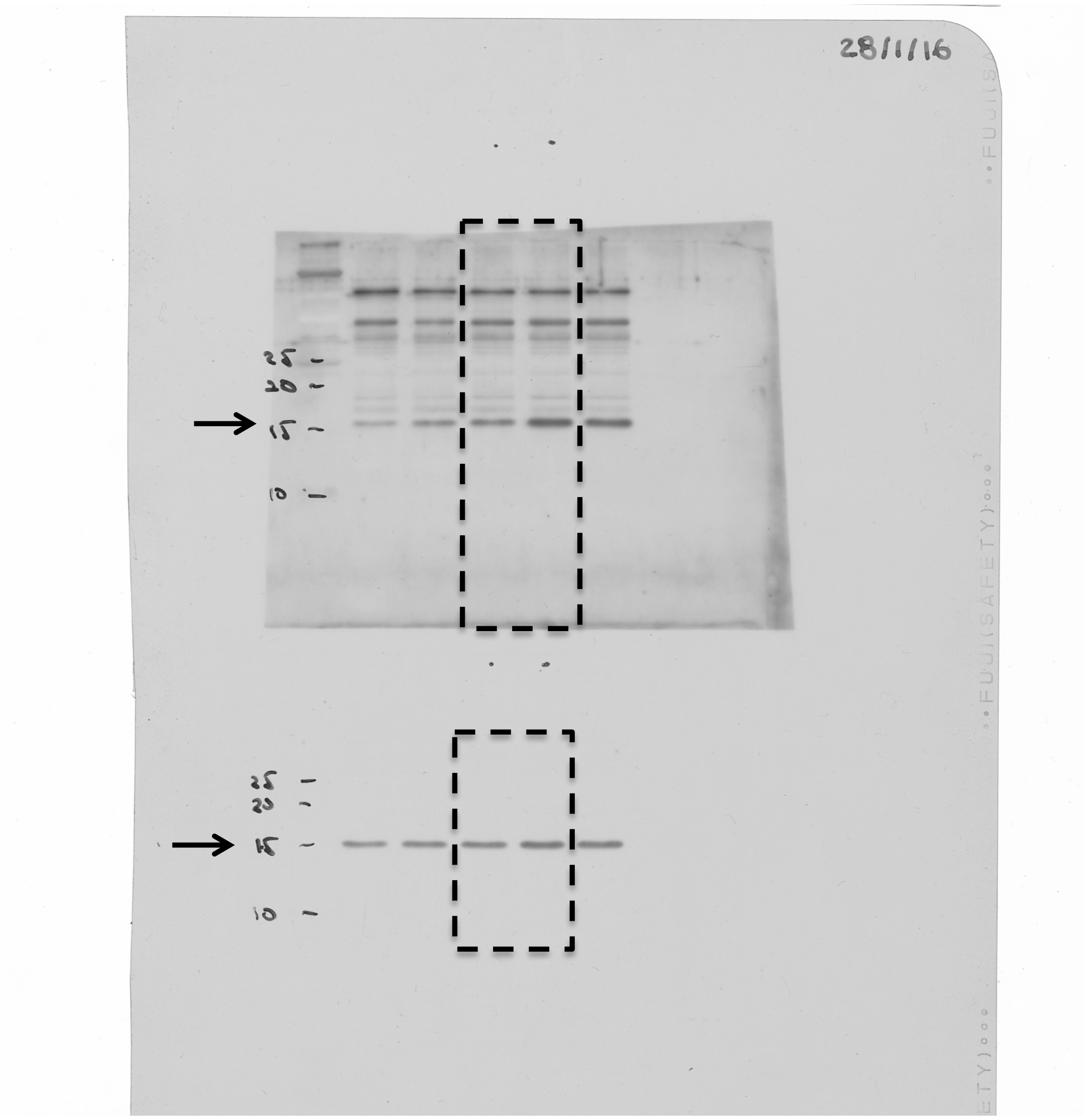

Fig. 7; panel A (dKDM5-HA; α tubulin)

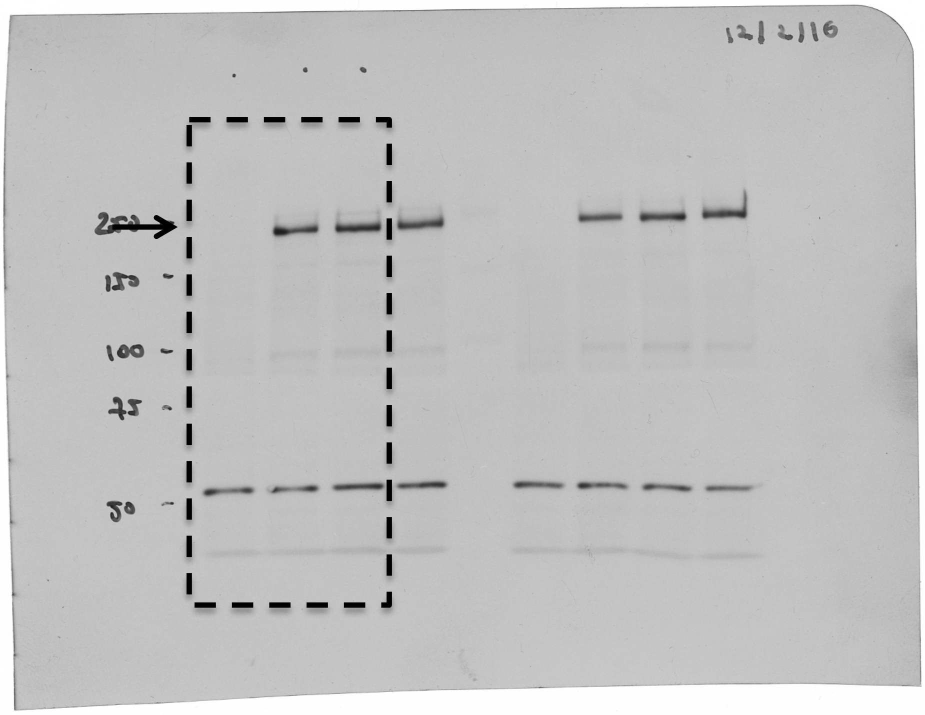

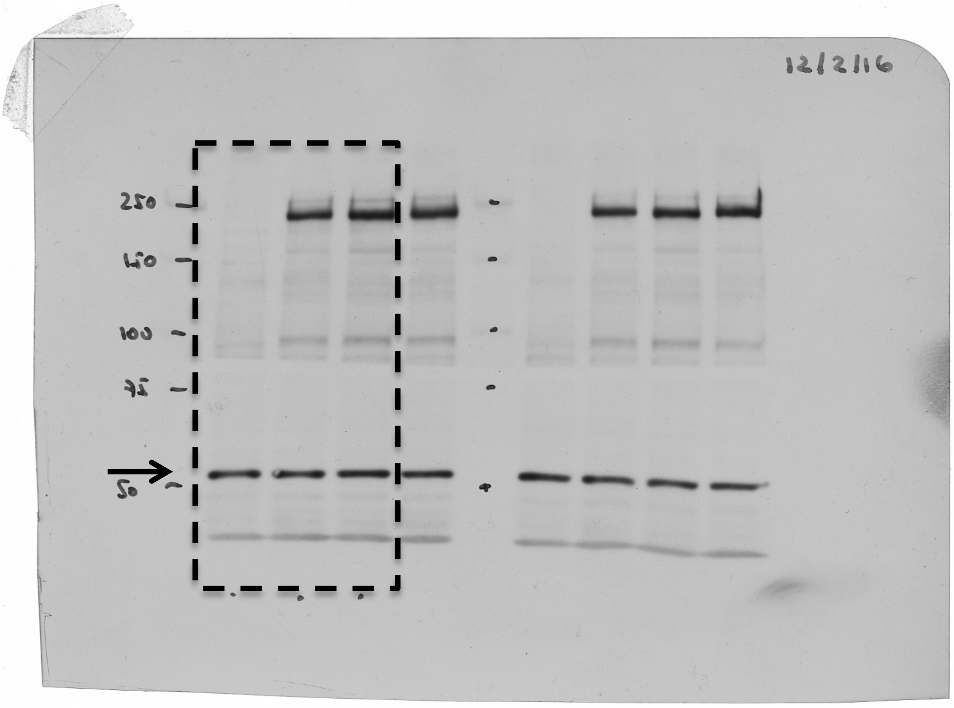

Sup. Fig. 3; panel A (dKDM5; α tubulin)

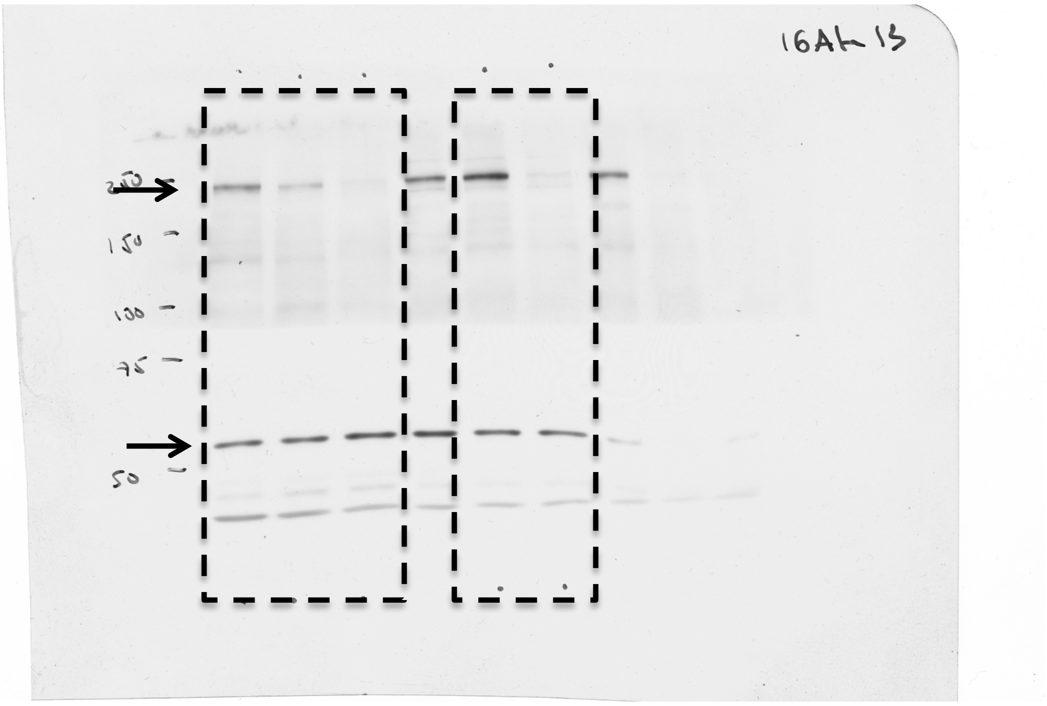

Sup. Fig. 5; panel A - RNAi (H3K4me3; H3)

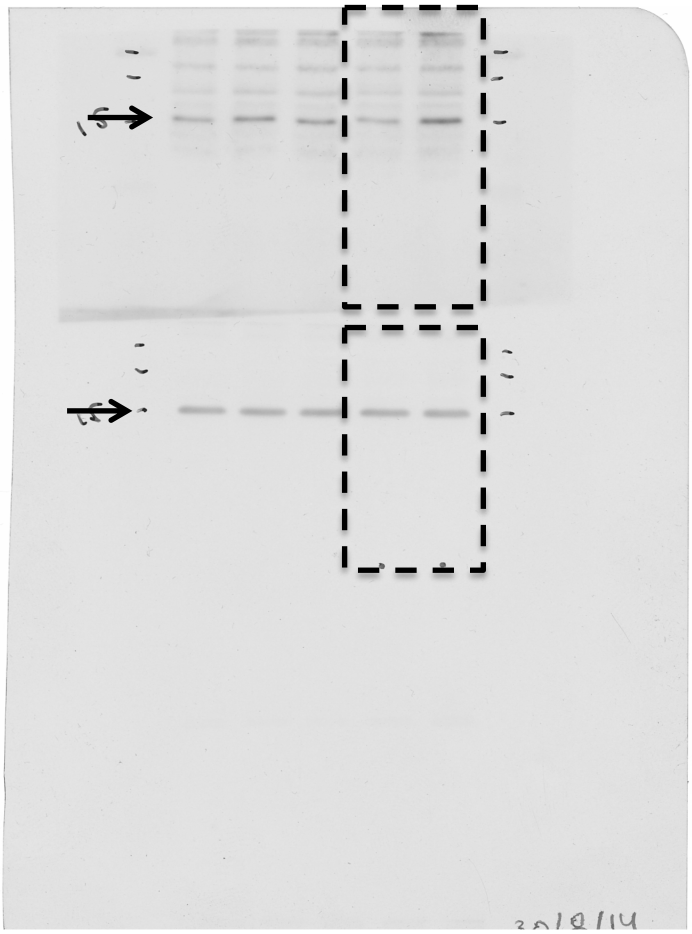

Sup. Fig. 5; panel A - *dkdm5*$^{-/-}$ (H3K4me3; H3)

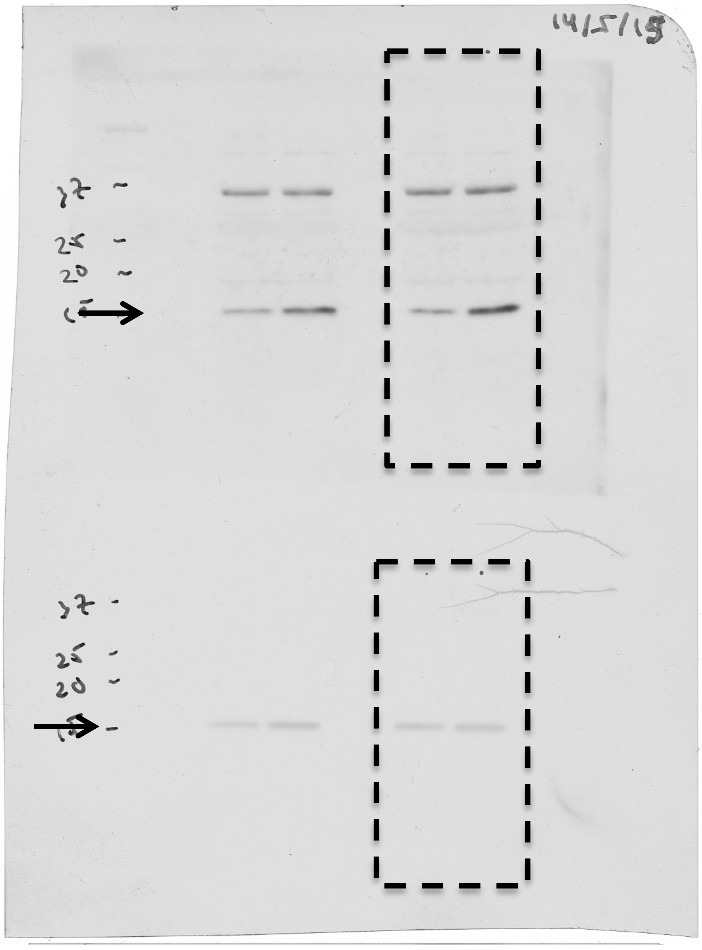

Supplementary Figure 14. Uncropped scans of all depicted protein blots.

Target Protein	Company	Fly Stock Number	Target Protein
Acf	Bloomington	31340	Acf
Art1	Bloomington	31348	Art1
Art4	Bloomington	36833	Art4
Art8	VDRC	v100228	Art8
Ash1	Bloomington	31050	Ash1
Ataxn7	VDRC	v103078	Ataxn7
Bap1	Bloomington	27061	Bap1
Bre1	Bloomington	28019	Bre1
Caf1	Bloomington	31714	Caf1
CG1539	Bloomington	35716	CG1539
CG1894	Bloomington	34925	CG1894
CG9293	VDRC	v102002	CG9293
dom	Bloomington	31054	dom
Eaf6	Bloomington	33904	Eaf6
eco	Bloomington	31343	eco
Elp3	Bloomington	35488	Elp3
enok	Bloomington	29518	enok
E(Pc)	Bloomington	28686	E(Pc)
EscL	VDRC	v108122	EscL
esc	Bloomington	31618	esc
G9a	Bloomington	24107	G9a
Gpp	VDRC	v110264	Gpp
Hdac3	Bloomington	31633	Hdac3
Hdac4	Bloomington	28549	Hdac4
Hdac6	Bloomington	31053	Hdac6
dKDM2	VDRC	v109295	dKDM2
dKDM5	Bloomington	28944	dKDM5
Mes4	Bloomington	34033	Mes4
mle	Bloomington	34864	mle
mof	Bloomington	31401	mof
Mrg15	Bloomington	35241	Mrg15
msl2	Bloomington	31627	msl2
msl3	Bloomington	35272	msl3
nej	Bloomington	35334	nej
nsl1	Bloomington	32561	nsl1
Ntmt	VDRC	v110351	Ntmt
pr-SET7	Bloomington	35322	pr-SET7
Rbbp5	Bloomington	42819	Rbbp5
Saf6	VDRC	v105259	Saf6
Setdb1	Bloomington	31352	Setdb1
sce	Bloomington	31612	sce
Sgf29	Bloomington	36637	Sgf29
Sir2	Bloomington	31636	Sir2
Sirt4	Bloomington	36588	Sirt4
trr	Bloomington	29563	trr
Trx	VDRC	v108122	Trx
UPSET	Bloomington	51447	UPSET
Usp36	Bloomington	27558	Usp36
Wdr82	Bloomington	32926	Wdr82
YL-1	Bloomington	31938	YL-1

Supplementary Table 1. RNAi lines used in the screen for regulators of the oocyte epigenome.

Antibodies used for epigenome characterization			
Histone Mark	Company	Product Code	Concentration
H2AK9ac	abcam	ab17346	1:500
H2BK5ac	abcam	ab61227	1:500
H2BK11ac	abcam	ab40975	1:500
H2BK12ac	abcam	ab1228	1:500
H2BK15ac	abcam	ab17351	1:500
H2BK16ac	abcam	ab40977	1:500
H2BK23ac	abcam	ab733830	1:500
H2BK120ub1	Active Motif	39624	1:500
H3K4me3	abcam	ab8580	1:500
H3K9ac	abcam	ab4441	1:500
H3K9me2	EMD Millipore	07-212	1:500
H3R17me2	abcam	ab8284	1:500
H3K27me3	abcam	ab6002	1:500
H3K36me	abcam	ab9048	1:500
H3K36me2	abcam	ab9049	1:500
H4R3me2	abcam	ab5823	1:500
H4K5ac	abcam	ab61236	1:500
H4K8ac	abcam	ab45166	1:500
H4K12ac	abcam	ab61238	1:500
H4K16ac	abcam	ab109463	1:500
H4K20me3	abcam	ab78517	1:500

Supplementary Table 2. Antibodies used for epigenome characterization.
